# Humic Acid Regulates Root Growth through ROS-Dependent Pathway and Hormone Signaling in Rice

**DOI:** 10.1021/acs.jafc.5c06288

**Published:** 2025-07-31

**Authors:** Andressa Fabiane Faria de Souza, Andrés Calderín García, José Nivaldo de Oliveira Sátiro, Brisa Ribeiro de Lima, Manlio Silvestre Fernandes, Ricardo Luiz Louro Berbara, Leandro Azevedo Santos

**Affiliations:** † Plant Nutrition Laboratory, Department of Soils, 67825 Federal Rural University of Rio de Janeiro (UFRRJ), Seropédica 23890-000, Rio de Janeiro, Brazil; ‡ Soil Biological Chemistry Laboratory, Department of Soils, 67825 Federal Rural University of Rio de Janeiro (UFRRJ), Seropédica 23890-000, Rio de Janeiro, Brazil

**Keywords:** biostimulant, plant development, scavenger, inhibitory effect, rice (*Oryza sativa* L.)

## Abstract

Humic acid (HA) enhances plant development, but the integration between reactive oxygen species (ROS) and hormone signaling in HA-induced root growth remains unclear. This study investigated the mechanisms of HA extracted from vermicompost on rice root development using ROS scavengers, hormone pathway inhibitors, and gene expression analyses. HA application increased root dry weight by 27% and lateral root length by 20%. ROS staining revealed higher O_2_^•–^ accumulation in root tips and H_2_O_2_ in the elongation zone. Treatments with SB, TIRON, DMTU, DPI, PCIB, and FLD suppressed HA-induced growth, indicating redox and hormonal modulation. HA also upregulated genes related to ROS signaling (*OsGPX3, CuZnSOD1, OsPRX112, BAS1*) and hormone signaling (*OsTOR, OsIAA11*). These findings support the idea that HA triggers a controlled oxidative signal that interacts with hormone pathways to regulate gene expression and root development. This ROS–hormone crosstalk reveals a key mechanism underlying HA bioactivity and reinforces its potential as a biostimulant for sustainable crop production.

## Graphical abstract

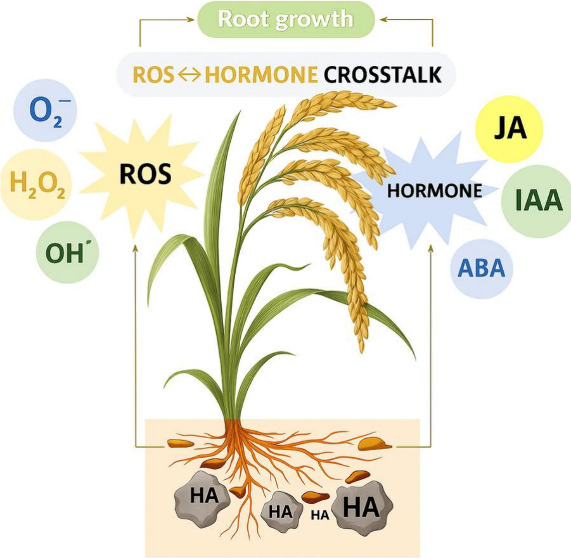

## Introduction

As the world population continues to grow, with projections indicating a potential increase to around 10.3 billion people in the mid-2080s,^1^ there will be an urgent need for much more effort and innovation to sustainably increase agricultural production. It is crucial to adopt sustainable agricultural practices that can support productive food systems through sound and sustainable management of soil, land, water, nutrients, and pest management, as well as the more extensive use of organic fertilizers.^2^

Due to the significant rise in global cereal consumption in recent years, rice production has increased worldwide. However, this increase is attributed to the extensive use of fertilizers, mainly nitrogen-based, leading to significant rises in production costs and environmental impacts.^3^ In this context the use of plant biostimulants is a promising and environmental-friendly technique to improve the ecological sustainability of crop production through a significant reduction of fertilizers and pesticides.^4–6^ The global biostimulants market reached 4.3 billion dollars in 2024 and is expected to grow over the next years, reaching 7.6 billion dollars by 2029, with an annual growth rate of around 12% from 2024 to 2029.^7^

Among biostimulant substances, humic acids (HA), particularly those derived from vermicompost, have shown promising effects on plant growth, including stimulation of root development, improved nutrient use efficiency, and enhanced tolerance to abiotic stress.^8–10^ Several studies have identified physiological and biochemical responses triggered by HA in different crops. For example, it was reported that the application of 20 mg·L^–1^ of HA from cattle manure vermicompost increased the number of lateral roots in maize seedlings by 40%.^11^ Similarly, another study reported that 1 mg·L^–1^ organic C of HA elevated indole-3-acetic acid (IAA) levels in *Arabidopsis* roots by 18%, promoting root elongation.^12^ HA treatment with 100 mg·L^–1^ organic C results an increase of ABA root concentration by 66% in cucumber.^13^ These effects have been partially attributed to hormonal modulation, especially of auxin and abscisic acid (ABA).

In parallel, HA has been shown to influence reactive oxygen species (ROS) dynamics, such as superoxide anion (O_2_^•–^) and hydrogen peroxide (H_2_O_2_), which function as key signaling molecules in plant development. It was demonstrated an increased in O_2_^•–^ and H_2_O_2_ accumulation in rice roots treated with HA (40 mg·L^–1^ organic C), alongside changes in root morphology.^14^ However, ROS modulation by HA appears to depend on both the chemical structure of the HA and the plant’s physiological condition, especially under stress.

Despite these findings, the molecular mechanisms behind HA-mediated root growth, particularly those involving the crosstalk between ROS and hormonal signaling, remain poorly understood. Few studies have directly tested how ROS accumulation interacts with hormone-related pathways in the presence of HA under controlled experimental conditions.

In this study, we investigated the effects of HA extracted from cattle manure vermicompost on rice (*Oryza sativa* L.) root development. We applied specific ROS scavengers and hormone pathway inhibitors, combined with morphological, histochemical, and gene expression analyses. Our working hypotheses are that

(i) HA promotes root growth through oxidative signaling regulation;
(ii) HA promotes root growth through hormonal signaling regulation;
(iii) HA triggers a combined ROS–hormone signaling interaction that drives root development.

## Materials and Methods

### Plant Material and Growth Conditions

Seeds of rice (*Oryza sativa* L. cv Nipponbare) were germinated in 3 L pots containing deionized water in a control growth chamber. At the time of germination, a nutrient solution was supplied with a modified ^1^/_4_ total ionic strength (IS) Hoagland solution^15^ containing 2 mM N (1.5 mM N- NO_3_^–^ and 0.5 mM N-NH_4_^+^). At 3 DAG (days after germination) the solution was replaced by the nutrient solution with a modified ^1^/_2_ IS Hoagland solution containing 2 mM N. At 5 DAG, 8 seedlings of the same size were transferred to each 50 mL falcon tubes containing a nutrient solution with a modified ^1^/_2_ IS Hoagland solution containing 2 mM N for better plant adaptation. All experiments were performed in a growth chamber under a light intensity of around 318–330 μmol m^–2^ s^–1^, a 14/10 h (h) light/dark photoperiod, 75% relative humidity, and a 28 °C day/24 °C night temperature, in the Department of Soil Sciences of the Federal Rural University of Rio de Janeiro (UFRRJ). The pH of the nutrient solutions was held at 5.8 and did not change significantly during the experiment and the nitrogen source was calcium nitrate and ammonium nitrate.

### Humic Acid Material and Treatments

Humic acids (HA) from vermicompost were extracted and purified following the method of International Humic Substances Society (IHSS) (https://humic-substances.org/isolation-of-ihss-soil-fulvic-and-humic-acids/). Briefly, the samples were stirred for 16 h in a 0.1 M KOH solution under a nitrogen atmosphere, using a 1:10 (w/v) ratio. After centrifugation and filtration, HA were isolated by acidification at pH 1.5 using 6 M HCl, which led to their precipitation. The resulting HA precipitate was then treated for 24 h with a mixture of HCl/HF and deionized water (1:1:98, v/v/v) to remove inorganic contaminants. Following thorough washing with distilled water to eliminate residual acid, the HA was transferred to a SPECTRA/POR dialysis membrane (10 kDa cutoff), frozen at–80 °C, and subsequently lyophilized and stored in a desiccator.

The chemical characterization was performed by using Fourier transform infrared spectroscopy (FTIR) and carbon-13 cross-polarization-magic angle spinning nuclear magnetic resonance (^13^C–CP/MAS NMR) spectroscopy. Details of the procedure were described previously.^16,17^ The relative amount of structures to these NMR intervals was used to calculate the percentage of aromaticity and aliphaticity.^18,19^ A summary of data related to the characterization of HA is presented in Supporting Information (Figures S1 and S2; Table S1).

To establish the optimal concentration of HA, the following concentrations were evaluated in the dose–response curve: 0 (control plants), 2, 5, 10, 30, 50, 80, 100, and 150 mg L^–1^ of organic carbon (C) (the concentrations were based on a previous experiment).^20^ The humic acids were solubilized in a 1 M KOH solution before being dissolved in a nutrient solution. The pH of the final nutrient solution was adjusted to 5.8.

The experimental design was entirely randomized with four biological replicates for each concentration (nine HA concentrations × four biological replicates, each biological replicate consisted of an average value of eight seedlings; *n* = 288). Two applications of HA were made at 6 and 9 DAG. In each application, 50 mL of freshly prepared nutrient solution containing the respective HA concentration was added to each Falcon tube. Plants were harvested at 12 DAG, 3 h after the start of the light period to minimize diurnal variations, for further analysis: shoot and root dry weight as well as measurement of root system. The concentration of 80 mg L^–1^ of organic carbon (C) of HA was detected as appropriated from the dose–response curve. A summary of data related to the optimal concentration of HA is presented in Supporting Information (Figure S3).

### Treatments with Scavengers and Inhibitors

To determine scavengers/inhibitors concentration that results in impaired root growth, without, however, causing severe toxicity to the plants, the following concentrations of sodium benzoate (SB); 4,5-dihydroxy-1,3-benzene disulfonic acid (TIRON); *N*,*N*′-dimethylthiourea (DMTU); diphenyleneiodonium chloride (DPI); 2-(*p*-chlorophenoxy)-2-methylpropionic acid (PCIB) and fluridone (FLD) were evaluated based on previous studies that used these compounds in rice or other model plants under similar experimental conditions.

After growing plants in the conditions described in the section Plant Material and Growth Conditions, 9 days after germination the following treatments were carried out to the dose–response curve:

(i) Sodium benzoate (SB) (HO* scavenger): 0 (control plants), 0.05, 0.5, 1, 1.5, 3, 5, 10, and 50 mM L^–1^ of SB.^21,22^ HO*: hydroxyl radical. Nine concentrations were established (*n* = 288).
(ii) 4,5-Dihydroxy-1,3-benzene disulfonic acid (TIRON), (O_2_^•–^ scavenger): 0 (control plants), 50, 100, 300, 500, 800, 1000, 1500, and 2000 μM L^–1^ of TIRON.^23,24^ O_2_^•–^: superoxide anion. Nine concentrations were established (*n* = 288).
(iii) *N*,*N*′-Dimethylthiourea (DMTU) (H_2_O_2_ scavenger): 0 (control plants), 0.5, 1, 2.5, 5, 10, 20, 50, and 100 mM L^–1^ of DMTU.^23,25,26^ H_2_O_2_: hydrogen peroxide. Nine concentrations were established (*n* = 288).
(iv) Diphenyleneiodonium chloride (DPI) (NADPH oxidase inhibitor): 0 (control plants), 0 (control plants with solvent), 1, 5, 10, 25, and 30 μM L^–1^ of DPI.^24–27^ NADPH: nicotinamide adenine dinucleotide phosphate. Seven concentrations were established (n = 224).
(v) 2-(*p*-Chlorophenoxy)-2-methylpropionic acid (PCIB) (auxin action inhibitor): 0 (control plants), 0 (control plants with solvent), 5, 10, 25, 50, 100, and 200 μM L^–1^ of PCIB.^28,29^ Eight concentrations were established (*n* = 256).
(vi) Fluridone (FLD) (ABA biosynthesis inhibitor): 0 (control plants), 0 (control plants with solvent), 5, 10, 25, 50, 100, 200 μM L^–1^ of FLD.^30–32^ ABA: abscisic acid. Eight concentrations were established (*n* = 256).

The experimental design was entirely randomized with four biological replicates for each concentration, and each biological replicate consisted of an average value of eight seedlings. Experiments with each scavenger/inhibitor were performed separately. The inhibitors DPI, PCIB and FLD were prepared in dimethyl sulfoxide (DMSO) and the scavengers SB, TIRON and DMTU were prepared in deionized water. The inhibitors were dissolved in DMSO to make 1,000× stock solution. Control plants for each experiment received the same corresponding amounts of solvent used to prepare the different inhibitors. Plants were harvested at 12 DAG, 3 h after the start of the light period to minimize diurnal variations, for further analysis: shoot and root dry weight, as well as measurement of root system.

The concentration of scavengers/inhibitors employed in the experiments was SB: 3 mM L^–1^; TIRON: 800 μM L^–1^; DMTU: 5 mM L^–1^; DPI: 5 μM L^–1^; PCIB: 25 μM L^–1^; FLD: 50 μM L^–1^. A summary of data related to the concentration of scavengers/inhibitors is presented in Supporting Information (Figures S4 to S9).

To analyze the residual effect of ROS scavengers and hormone inhibitors, a time-course experiment was conducted using the concentrations based on a previous result with the aim of evaluating the effect of the scavenger/inhibitors over time and thus answer the question of how long after renewing the nutrient solution the effect of the scavenger/inhibitor on the plant would still be possible to detect.

At 6 DAG three plant treatments were applied to the nutrient solution: (1) control plants grown in nutrient solution for the duration of the experiment; (2) and treated plants with (+) scavenger/inhibitor, separately. At 9 DAG a new nutrient solution was supplied, and three treatments were applied to the nutrient solution: (1) control plants grown in nutrient solution for the duration of the experiment; (2) plants without (−) the scavenger/inhibitor in nutrient solution (initial treatment with scavenger/inhibitor for a period of 3 days and supplied with a new nutrient solution after removing the scavenger/inhibitor solution); (3) and treated plants with (+) scavenger/inhibitor-treated plants (initial treatment with scavenger/inhibitor for a period of 3 days and supplied with a new nutrient solution containing scavenger/inhibitor solution) (Figure 1). The experimental design was entirely randomized with four biological replicates for each treatment (three plant treatments), and each biological replicate consisted of an average value of eight seedlings (*n* = 96). Experiments with each scavenger or inhibitor were performed separately. The following parameters were measured at 24, 48, and 72 h after treatment: shoot and root dry weight, as well as measurement of the root system.

**Figure 1:**
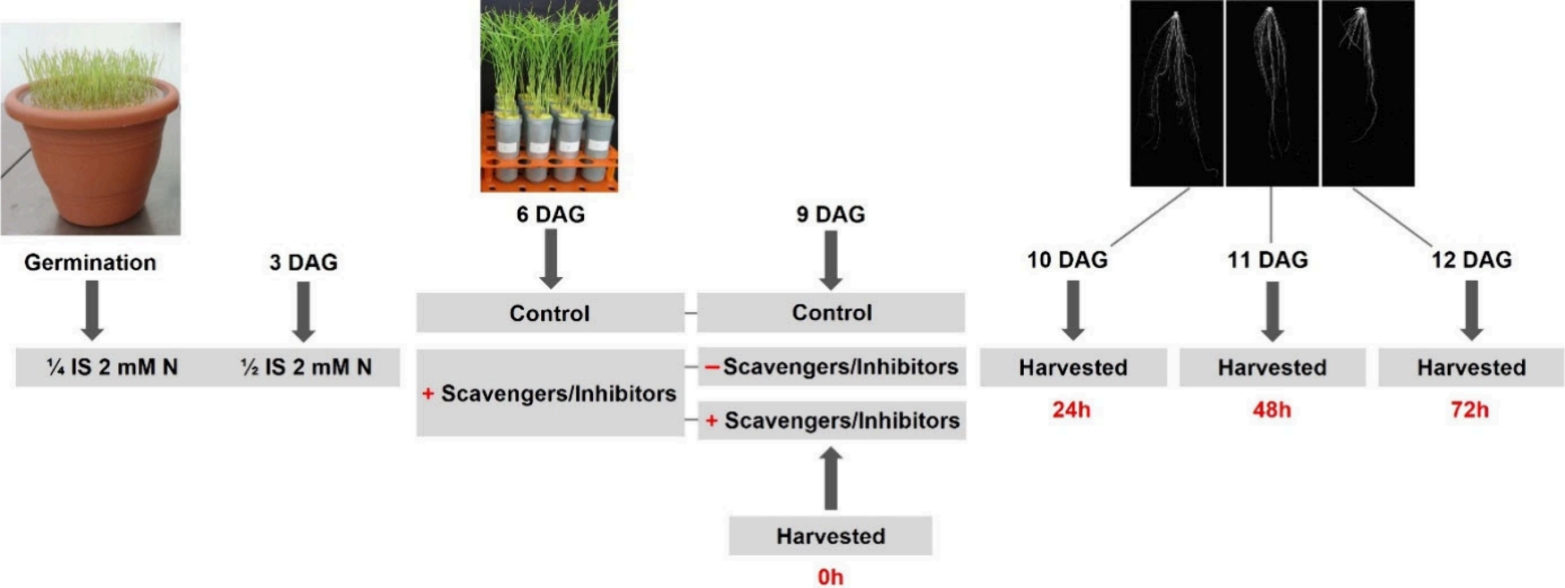
Schematic overview of the time-course treatments. The timeline shows germination, treatment initiation (6 DAG), and harvesting at 0, 24, 48, and 72 h after the treatment. (+) scavenger/inhibitor-treated plants, (−) plants without the scavenger/inhibitor. DAG: days after germination; IS: ionic strength.

### Rice DR5::GUS Histochemical Bioassay of ROS

A bioassay was performed using the DR5::GUS transgenic rice construct, a synthetic auxin-responsive promoter that directs the expression of the reporter gene *gus* (β-glucuronidase). Three-day-old (3–4 cm) DR5::GUS transgenic rice plants, cultivated with a modified ^1^/_4_ total ionic strength (IS) Hoagland solution^15^ containing 0,5 mM N, were treated: (1) control plants grown in nutrient solution for the duration of the experiment; (2) HA-treated plants (80 mg·L^–1^ of organic carbon added to the nutrient solution); (3) HA → scavenger or inhibitor combined treatment (initial treatment of 80 mg·L^–1^ of organic carbon for a period of 30 min and addition of scavenger or inhibitor to a new nutrient solution after removing the HA solution). The experimental design was entirely randomized with four biological replicates for each treatment (three treatments), and each biological replicate consisted of an average value of eight seedlings (*n* = 96). Experiments with each scavenger or inhibitor were performed separately.

After 24 h, the histochemical staining was performed. Superoxide anion (O_2_^•–^) was visualized using a blue solution of p-nitro tetrazolium (NBT) and hydrogen peroxide (H_2_O_2_) was visualized as a brown color that was caused by the polymerization of 3,3-diaminobenzidine (DAB) as described previously.^33^ β-glucuronidase activity (GUS) was visualized as a blue color as described previously.^34^

### Validation of the Experimental Model

To investigate the mechanisms responsible for the direct effect of HA in promoting root growth, the following experimental models were proposed in this study:

(i) Evaluation of the relevance of the effects of HA in promoting root and shoot growth, through the application of HA and subsequent application of the scavenger/inhibitor.
(ii) Evaluation of the relevance of the effects of HA in promoting root and shoot growth, through the application of the scavenger/inhibitor and then application of the HA.

In this experiment, three plant treatments were applied at 6 DAG to the nutrient solution: (1) control plants grown in nutrient solution for the duration of the experiment; (2) HA-treated plants (80 mg·L^–1^ of organic carbon added to the nutrient solution); (3) and scavenger/inhibitor-treated plants. At 9 DAG a new nutrient solution was supplied and five treatments were applied to the nutrient solution: (1) control plants grown in nutrient solution for the duration of the experiment; (2) HA-treated plants (80 mg·L^–1^ of organic carbon added to the nutrient solution); (3) HA → scavenger or inhibitor combined treatment (initial treatment of 80 mg·L^–1^ of organic carbon for a period of 72 h (3 days) and addition of scavenger or inhibitor to a new nutrient solution after removing the HA solution); (4) Scavenger or Inhibitor-treated plants (treatment with scavenger or inhibitor for a period of 3 days and supplied with a new nutrient solution containing scavenger/inhibitor solution); (5) Scavenger or Inhibitor → HA combined treatment (initial treatment of scavenger/inhibitor for a period of 72 h (3 days) and addition of 80 mg·L^–1^ of organic carbon from HA to a new nutrient solution after removing the scavenger or inhibitor solution). The experimental design was entirely randomized with four biological replicates for each treatment, and each biological replicate consisted of an average value of eight seedlings (*n* = 160). Experiments with each scavenger or inhibitor were performed separately. The following parameters were measured at 12 DAG (72 h after treatment): shoot and root dry weight, as well as measurement of root system, and roots were frozen in liquid nitrogen and stored in a −80 °C freezer for RNA extraction.

For root morphology analysis, the roots were scanned at 600 dpi with an Epson Expression 10000XL scanner. The images were analyzed using the WinRhizo *Arabidopsis* scanner-based image analysis system software (Regent Instruments, Montreal, QC, Canada, 2012b) at a setting of 600 dpi (dots per inch). The following parameters were determined: total root length (mm), total average diameter (mm), total root surface area (mm^2^), total root volume (mm^3^), total number of tips, and length of lateral roots (LR, mm).

### RNA Isolation and RT- qPCR Analysis

The expression of 14 genes involved in hormonal signaling pathways and the reactive oxygen species (ROS) metabolism was investigated in control, HA-treated and scavenger or inhibitor-treated seedlings. In the ROS signaling the expression pattern of *BAS1* (*2-Cys peroxiredoxin 1*), *OsGPX3* (*glutathione peroxidase 3*), *OsPRX112* (*peroxidase isoform 2*), *CuZnSOD1* ([*Cu–Zn*] *superoxide dismutase 1*), in hormonal pathway the expression of genes such as *OsTOR* (*Target of Rapamycin*) and *OsIAA11* (*3-Indolacetic acid 11*), physiological responses linked to root growth and N uptake such as *OsA7* (isoform of plasma membrane H^+^-ATPase, *Oryza sativa 7*), *OsNRT2.1* (*nitrate transporter 2.1*), regulatory proteins (protein kinases) involved in calcium signaling pathways as *OsCPK7* (*calcium-dependent protein kinase gene family*–CDPK), *OsTPC1* (*two-pore channel 1*), effects on regulation of vesicle transport like *SEC1B* (for secretion–family of protein secretion and transport), *TOM1* (*transporter of mugineic acid 1*), primary metabolism as *HXK5* (*hexokinase-5*) and *PHS1* (*phosphorylase 1*) was carry out the gene expression study.

Approximately 80–100 mg of tissue sample was used for total RNA extraction as described previously.^35^ RNA quality and concentration were verified by A_260/230_ and A_260/280_ proportion, with the ratio between 1.9 and 2.1, using Nanodrop (Thermo Scientific) and the RNA integrity was verified by agarose (1%) gel electrophoresis. One μg of total RNA was treated with DNase I Amplification grade (Sigma-Aldrich) and DNase-treated RNA was converted to cDNA, using High-Capacity cDNA Reverse Transcription Kits 200 reactions (Applied Biosystems) according to the manufacturer’s instructions. The RT-q PCR analysis was performed in a StepOne Plus Real-Time PCR System (Applied Biosystems) using the SYBR Green PCR Master Mix. To determine the optimal endogenous control, the Normfinder software was used, and the most stable endogenous control was selected. The relative gene expression data was analyzed using the delta–delta comparative Ct method (2^–ΔΔ*C*^_T_)^36^ using the *OsUBQ5* (Ubiquitin) as endogenous control^37^ and transcript levels were normalized to the control. All primers are listed in Supporting Information (Table S2).

### Statistical Analysis

Quadratic regression was fitted to the dose–response curve. The normal distribution of the treatments were analyzed using the Shapiro–Wilk test data normality (*p* > 0.05) and homoscedasticity of variances using Levene’s test (*p* > 0.05). Analysis of variance (ANOVA) and Tukey’s posthoc test at 5% probability (*p*-value ≤ 0.05) was used to perform mean comparisons. The effects of the variables were verified by F test (5% probability). Statistical analyses were performed using Real Statistics, a free add-in for Excel.

## Results

### Residual Effect of ROS Scavengers and Hormone Inhibitors

The results show that 72 h after removal of the scavenger solutions, SB and TIRON (Figure 2a,b), the root dry weight reduced only 13% and 5%, respectively, compared to control, without scavenger solutions. Consequently, plant growth was restored due to the reduction in their effects, showing a short residual effect of 24 h. For scavenger or inhibitor solutions, DMTU (Figure 2c), DPI (Figure 2d), PCIB and FLD (Figure 3a,b, respectively), it has been shown that 72 h after removal of the scavenger solutions still have an inhibitory effect and decreased significantly the root growth to 61%, 34%, 39%, and 55%, respectively, compared to control. These finds are similar to the treatments that keep the scavenger/inhibitors solution permanently, showing a residual effect during the trial period of at least 72 h.

**Figure 2:**
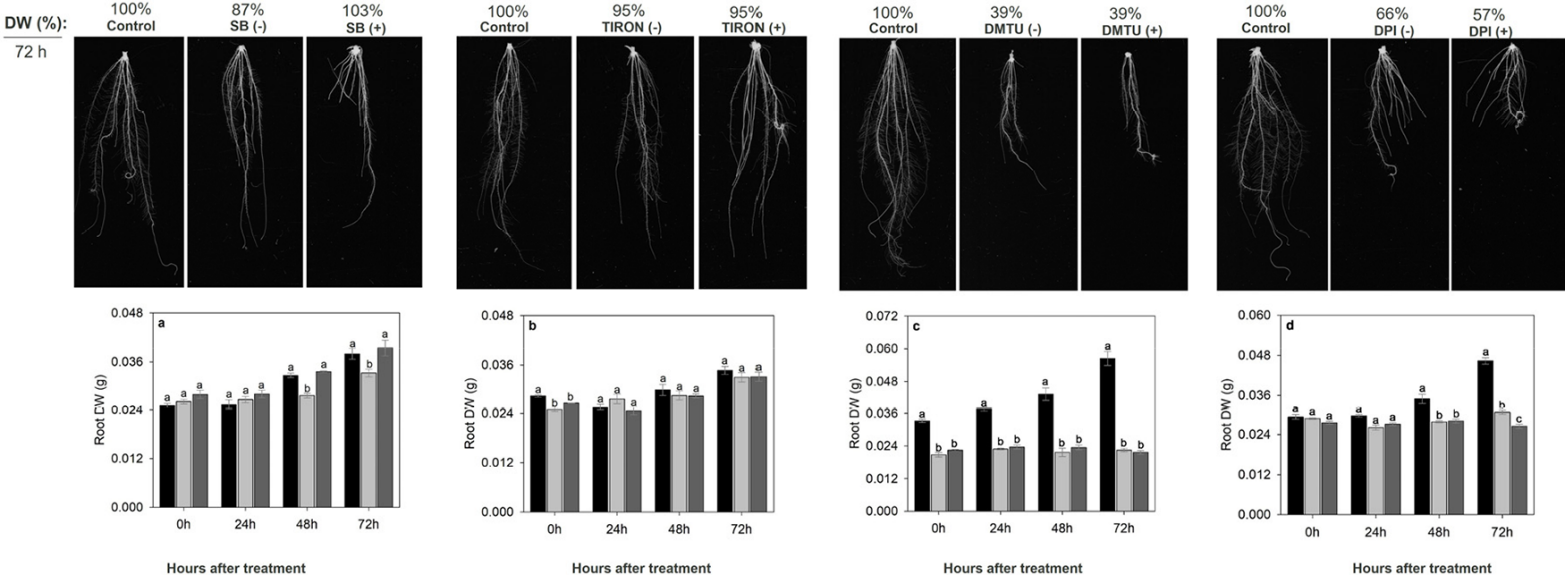
Scavengers time-course. The plants were harvested at 0, 24, 48, and 72 h (hours) after the treatment. (a) Root biomass of rice plants during exposure to SB (HO* scavenger). (b) Root biomass of rice plants during exposure to TIRON (O_2_^•–^ scavenger). (c) Root biomass of rice plants during exposure to DMTU (H_2_O_2_ scavenger). (d) Root biomass of rice plants during exposure to DPI (NADPH oxidase inhibitor). DW (g): dry weight in grams. (−): plants without the scavenger; (+): scavenger-treated plants. Bars indicate the four biological replicates standard error. Different letters above the bars represent significant differences according to Tukey’s test (*p*-value ≤ 0.05).

**Figure 3:**
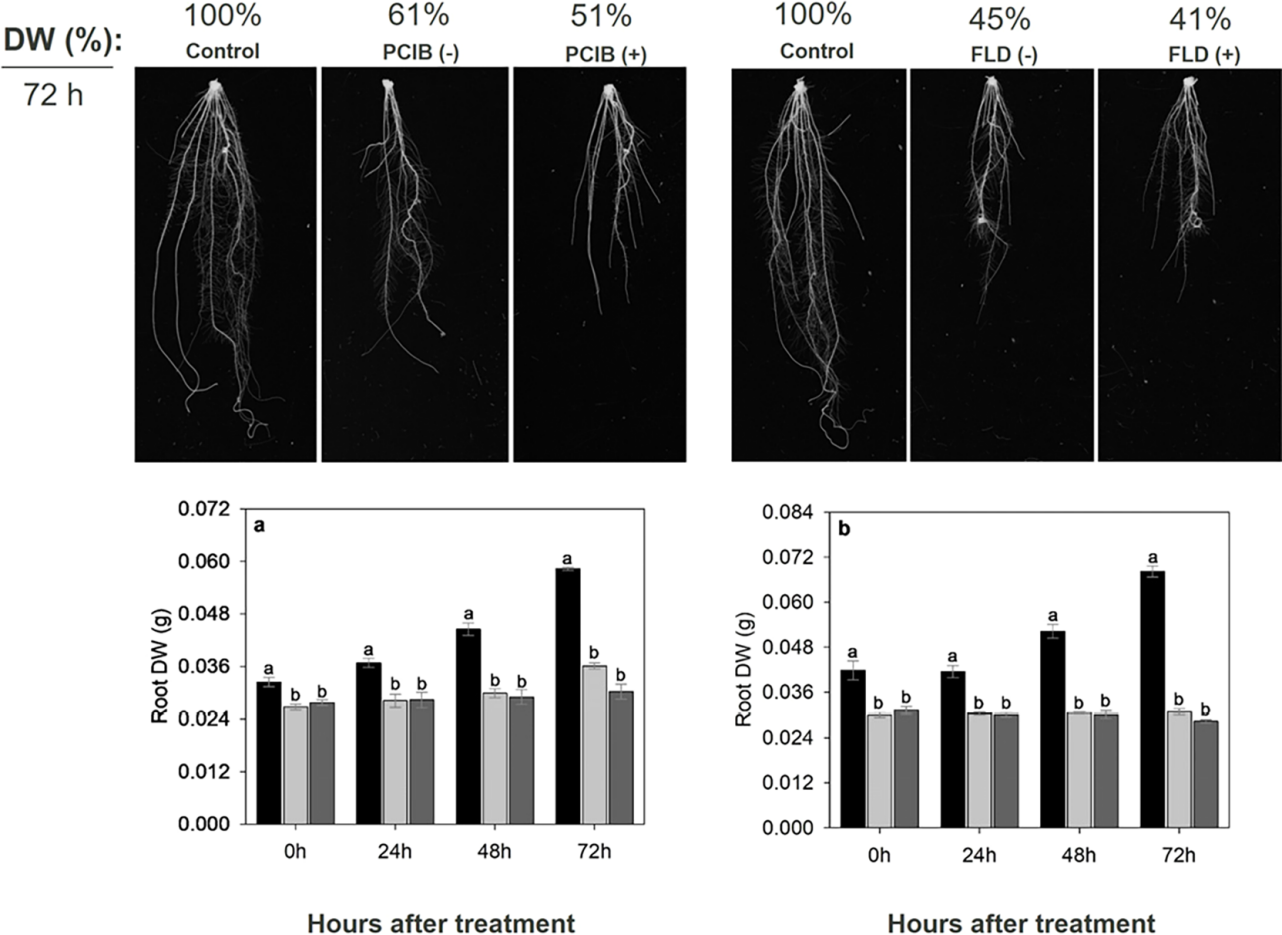
Inhibitor time-course. The plants were harvested at 0, 24, 48, and 72 h (hours) after the treatment. (a) Root biomass of rice plants during exposure to PCIB (auxin action inhibitor). (b) Root biomass of rice plants during exposure to FLD (ABA biosynthesis inhibitor). DW (g): dry weight in grams. (−): plants without the scavenger; (+): scavenger-treated plants. Bars indicate the four biological replicates standard error. Different letters above the bars represent significant differences according to Tukey’s test (*p*-value ≤ 0.05).

### HA Triggers the ROS and Auxin Signaling Pathways

Several studies using DR5::GUS construct have shown that the application of HA to the roots induced auxin responses.^12,38,39^ It was observed that at 24 h following the exposure to the treatments, the GUS expression pattern was different in HA-treated and scavengers/inhibitor-treated seedlings compared to the control (Figure 4a–c). To stain detection of ROS (O_2_^•–^) (Figure 4a) an accumulation was observed to be more intense in the root tips of both primary and lateral roots of HA-treated and inhibitor/scavengers-treated seedlings compared to the control. For H_2_O_2_ an accumulation of these ROS was observed in the region of elongation of the roots (Figure 4b), as well as to the GUS staining (Figure 4c).

**Figure 4:**
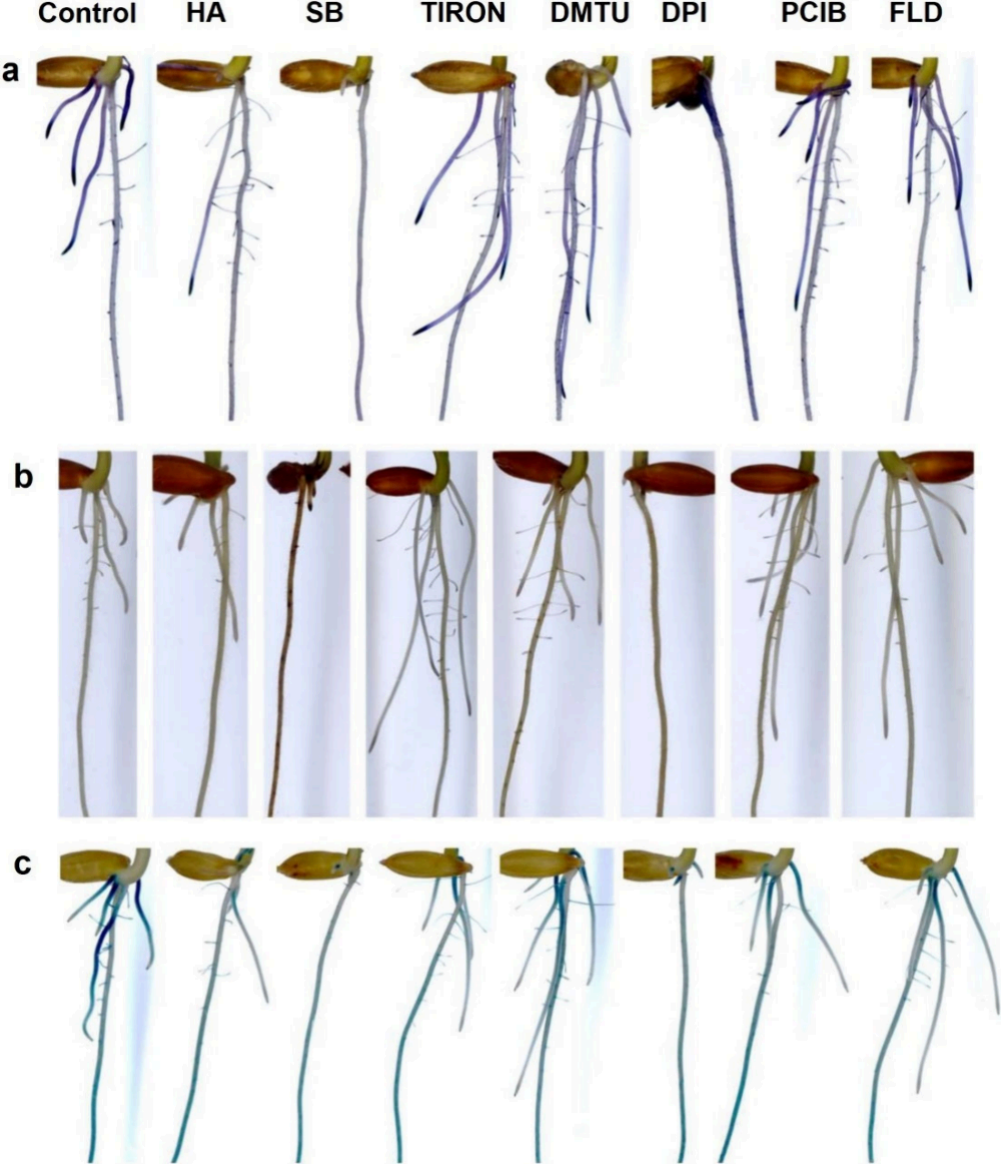
ROS staining detection at 3 DAG in transgenic rice root DR5::GUS. After 24 h, the histochemical staining was performed. (a) NBT staining to visualize superoxide anion (O_2_^•–^). (b) DAB staining to visualize hydrogen peroxide (H_2_O_2_). (c) β-Glucuronidase activity (GUS). SB: sodium benzoate (HO* scavenger). TIRON: 4,5-dihydroxy-1,3-benzene disulfonic acid (O_2_^•–^ scavenger). DMTU: *N*,*N*′-dimethylthiourea (H_2_O_2_ scavenger). DPI: diphenyleneiodonium chloride (NADPH oxidase inhibitor). PCIB: 2-(*p*-chlorophenoxy)-2-methylpropionic acid (auxin action inhibitor). FLD: fluridone (ABA biosynthesis inhibitor). HA: humic acid and was used 80 mg·L^–1^ of organic carbon. The concentration of scavengers/inhibitors employed in the experiments was SB: 3 mM L^–1^. TIRON: 800 μM L^–1^. DMTU: 5 mM L^–1^. DPI: 5 μM L^–1^. PCIB: 25 μM L^–1^. FLD: 50 μM L^–1^.

### Effects of HA to the Root and Shoot Growth-Promoting Effects by the Application of ROS Scavengers and Hormonal Inhibitors

In all the bioassays performed, the biostimulant effect of HA on root growth was verified, and this was associated with several events described below. To investigate the role of HA-mediated ROS accumulation in roots, a HO* scavenger (SB), O_2_^•–^ scavenger (TIRON), H_2_O_2_ scavenger (DMTU), NADPH oxidase inhibitor (DPI) were applied along with HA.

HA application increased root dry weight by 27% and lateral root length by 20% compared to the control. When the plants are first treated with HA and then transferred to SB treatment, significantly reduced root length by 24%, results in damage to root growth, with a completely block of HA action (Figure 5d,e). Furthermore, using the rice seedling staining detection bioassay, we verified that SB application caused an excessive increase in H_2_O_2_ (Figure 4b). Similarly, when plants are TIRON-treated after the HA treatment inhibited root and shoot growth by 9% compared to control (only nutrient solution), plant growth was at the level of plants treated only with TIRON for the duration of the experiment (Figure 5h,i).

**Figure 5:**
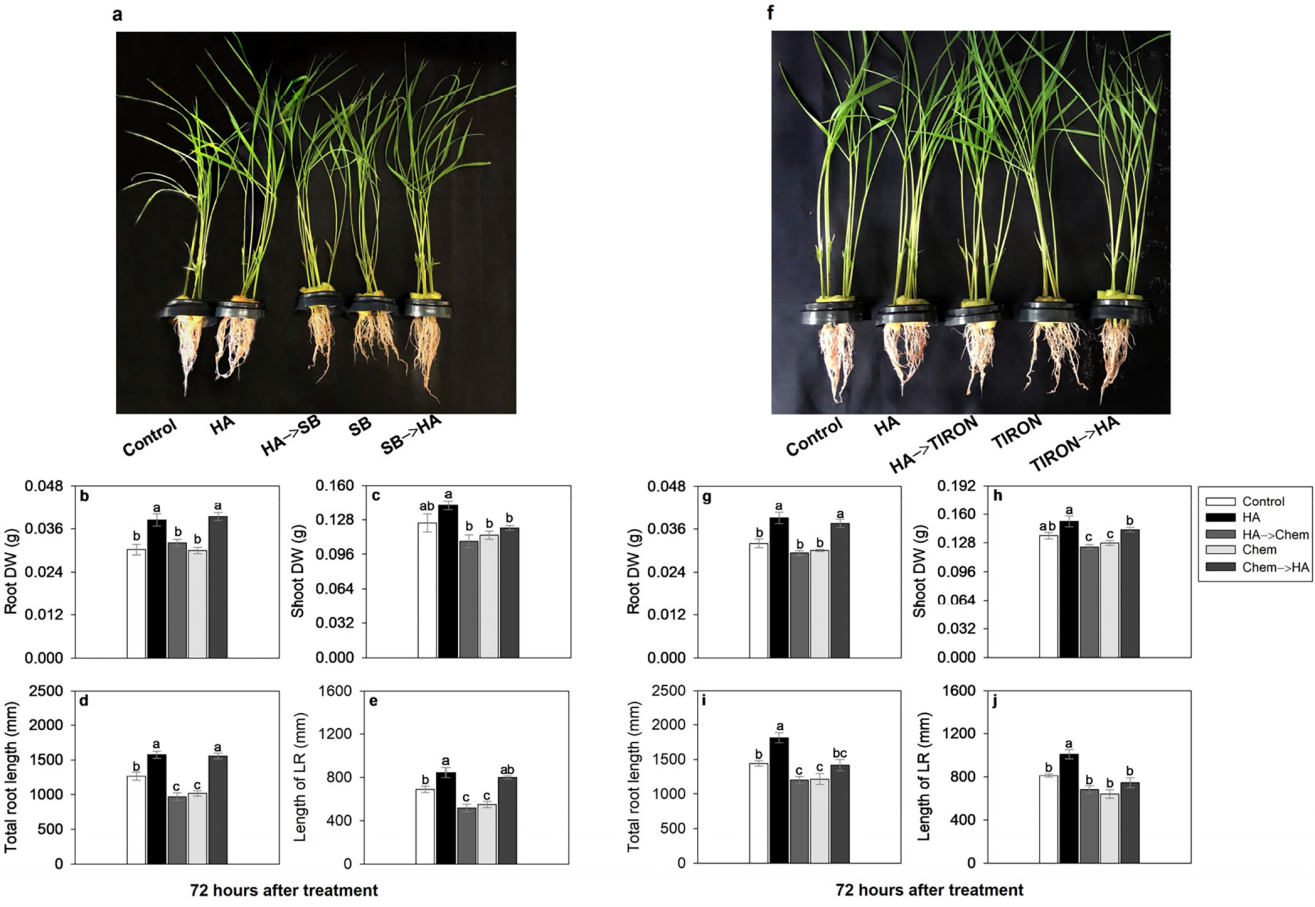
Effect of 80 mg·L^–1^ of organic carbon from HA, SB (HO* scavenger), and TIRON (O_2_^•–^ scavenger) on root and shoot development. Rice plants grown for 72 h in nutrient solution with HA and addition of SB (3 mM L^–1^) or TIRON (800 μM L^–1^). (a–e) Root and shoot biomass, and root morphology of rice plants during exposure to HA and SB. (f–j) Root and shoot biomass, and root morphology of rice plants during exposure to HA and TIRON. The analysis of root morphology was carried out using WinRhizo *Arabidopsis* software. SB: sodium benzoate. HO*: hydroxyl radical. TIRON: 4,5-dihydroxy-1,3-benzene disulfonic acid. O_2_^•–^: superoxide anion. HA: humic acid. DW (g): dry weight in grams. LR: lateral root. Chem: a chemical substance added to solution as an scavenger. Bars indicate the four biological replicates standard error. Different letters above the bars represent significant differences according to Tukey’s test (*p*-value ≤ 0.05).

It is important to note that when the plants are transferred from SB or TIRON treatment to HA, the root growth was restored at the level of plants treated with HA for the duration of the experiment (Figure 5b,g), which confirms no residual effect throughout the trial period after the scavenger was removed from the solution. Therefore, the treatment in which the plants are transferred from the SB and TIRON scavenger to the AH should not be considered.

The application of HA to plant roots resulted in improved shoot and root growth by 10% and 16%, respectively, after 72 h of treatment, while the addition of DMTU results in damage compared to control treatments (Figure 6a–e). The results showed that the addition of DMTU was able to reduce by 32% the HA biostimulant effect in the treatments. In contrast, when the plants are transferred from DMTU treatment to HA, the root growth was restored despite the residual effect already demonstrated for the DMTU (Figure 2c).

**Figure 6:**
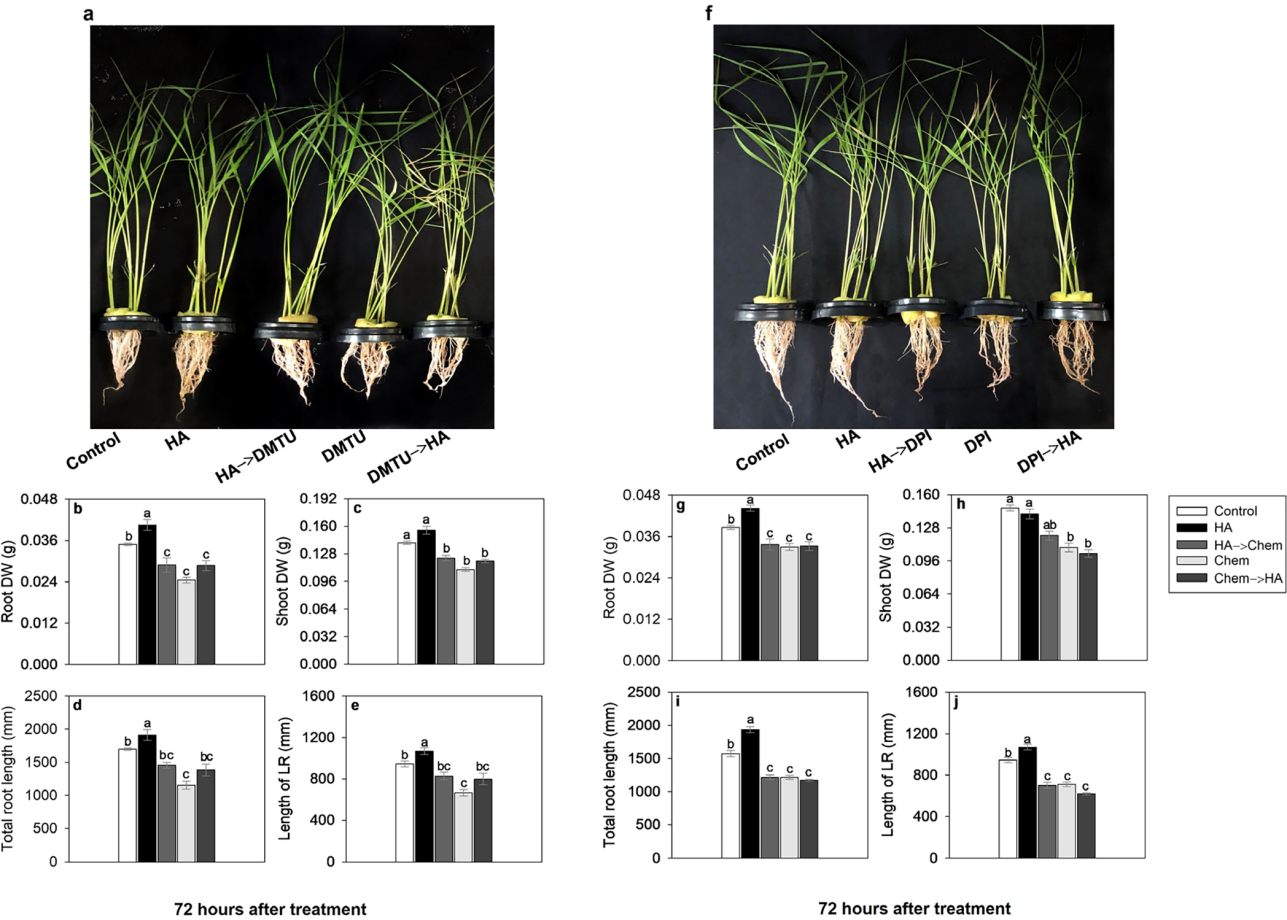
Effect of 80 mg·L^–1^ of organic carbon from HA, DMTU (H_2_O_2_ scavenger), and DPI (NADPH oxidase inhibitor) on root and shoot development. Rice plants grown for 72 h in nutrient solution with HA and addition of DMTU (5 mM L^–1^) or DPI (5 μM L^–1^). (a–e) Root and shoot biomass, and root morphology of rice plants during exposure to HA and DMTU. (f–j) Root and shoot biomass, and root morphology of rice plants during exposure to HA and DPI. The analysis of root morphology was carried out using WinRhizo *Arabidopsis* software. DMTU: *N*,*N*′-dimethylthiourea. H_2_O_2_: hydrogen peroxide. DPI: diphenyleneiodonium chloride. NADPH: nicotinamide adenine dinucleotide phosphate. HA: humic acid. DW (g): dry weight in grams. LR: lateral root. Chem: a chemical substance added to solution as scavenger (DMTU) or an inhibitor (DPI). Bars indicate the four biological replicates standard error. Different letters above the bars represent significant differences according to Tukey’s test (*p*-value ≤ 0.05).

In the presence of DPI, root dry weight decreased by 12%, total root length by 31%, and lateral root length by 29%, with similar reductions observed in both treatment sequences (HA→DPI and DPI→HA) (Figure 6g–j). It is significant to observe that when the plants are transferred from DPI treatment to HA, the residual effect of DPI inhibits any possible HA stimulatory effect. This indicates that the formation of O_2_^•–^, which is quickly converted to H_2_O_2_,^40^ is essential for root growth. Similarly, when the plants are first treated with HA and then transferred to DPI treatment results in damage to root growth, suggesting that DPI is capable of blocking any stimulatory effect of HA. This suggests the existence of a preferential pathway in the mechanism of HA’s action. Also, these data confirm that root growth promotion is a ROS-dependent mechanism, and the stimulatory effect of HA was dependent on O_2_^•–^ production. Once again, as with the use of DPI, the need for ROS O_2_^•–^/H_2_O_2_ for root growth is evident. Since O_2_^•–^ radical is important in root growth^46^ plants under the inhibitory effect of O_2_^•–^ production, due to the application of TIRON and DPI scavenger, had shorter and thicker roots, a reduction in surface area, root volume and fewer tips (Figure S10).

To understand the role of HA-mediated hormone signaling in roots, inhibitors of the auxin action (PCIB) and ABA biosynthesis (FLD) were applied along with HA. The application to plant roots of PCIB inhibited the shoot by 25% and root growth by 9% compared to control (Figure 7a–e). When the plants are transferred from HA to PCIB treatment, they have better root growth than those treated with PCIB only, despite the residual effect already demonstrated for PCIB (Figure 3a), suggesting that PCIB is not able to completely block the stimulatory effect of HA. However, when the plants are transferred from PCIB treatment to HA, the residual effect of PCIB inhibits any possible HA stimulatory effect.

**Figure 7:**
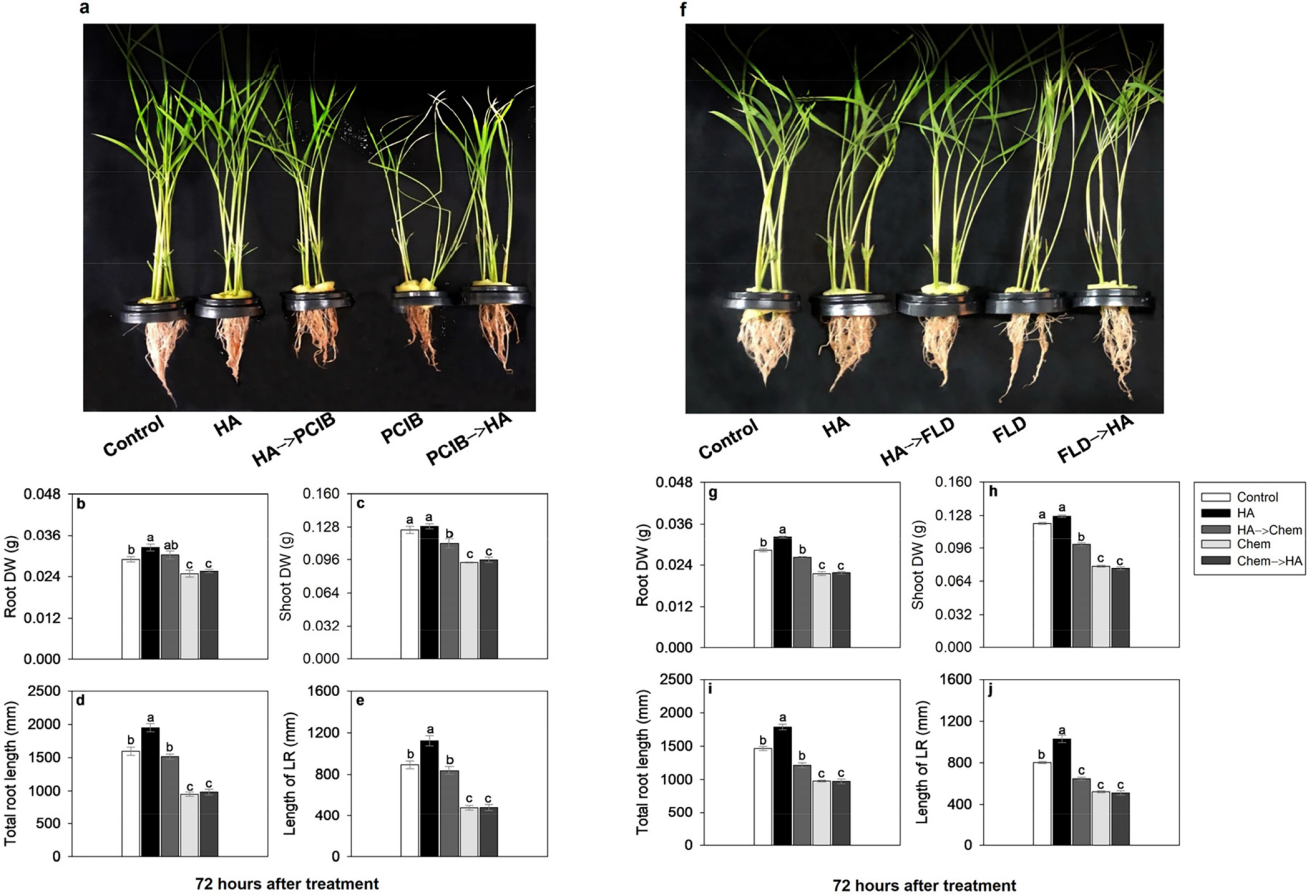
Effect of 80 mg·L^–1^ of organic carbon from HA, PCIB (auxin action inhibitor), and FLD (ABA biosynthesis inhibitor) on root and shoot development. Rice plants grown for 72 h in nutrient solution with HA and addition of PCIB (25 μM L^–1^) or FLD (50 μM L^–1^). (a–e) Root and shoot biomass, and root morphology of rice plants during exposure to HA and PCIB. (f–j) Root and shoot biomass, and root morphology of rice plants during exposure to HA and FLD. The analysis of root morphology was carried out using WinRhizo *Arabidopsis* software. PCIB: 2-(*p*-chlorophenoxy)-2-methylpropionic acid. FLD: fluridone. ABA: abscisic acid. HA: humic acid. DW (g): dry weight in grams. LR: lateral root. Chem: a chemical substance added to solution as an inhibitor. Bars indicate the four biological replicates standard error. Different letters above the bars represent significant differences according to Tukey’s test (*p*-value ≤ 0.05).

The application of FLD, exhibited a pattern similar to the application of PCIB, inhibiting the shoot by 40% and root growth by 30% compared to control (Figure 7f-j). This experiment showed that when the plants are transferred from HA to FLD treatment, they have better root growth than those treated with FLD only, despite the residual effect already demonstrated for the FLD (Figure 3b). Once again, as with the use of PCIB, FLD is not able to block completely the stimulatory effect of HA. On the other hand, when the plants are transferred from FLD treatment to HA, the residual effect of FLD inhibits any possible HA stimulatory effect.

### HA Increases the Expression of Genes Involved in ROS and Hormone Signaling Pathways That Are Essential for Root Growth

In addition to the results of shoot and root dry weights and root morphology of rice plants that are improved due to the stimulatory effect of HA, the expression of genes involved in ROS and hormone signaling pathways was also analyzed. HA-treated plants showed increases in the expression of *OsGPX3* (*glutathione peroxidase*) and *CuZnSOD1* ([*Cu–Zn*] *superoxide dismutase 1*) (Figure 8), and *BAS1* (*2-Cys peroxiredoxin 1*) (Figure S11), *OsPRX112* (*peroxidase isoform 2*) (Figure S12) genes compared to control plants 72 h after the HA treatment and the combinations HA and scavenger/inhibitor.

**Figure 8:**
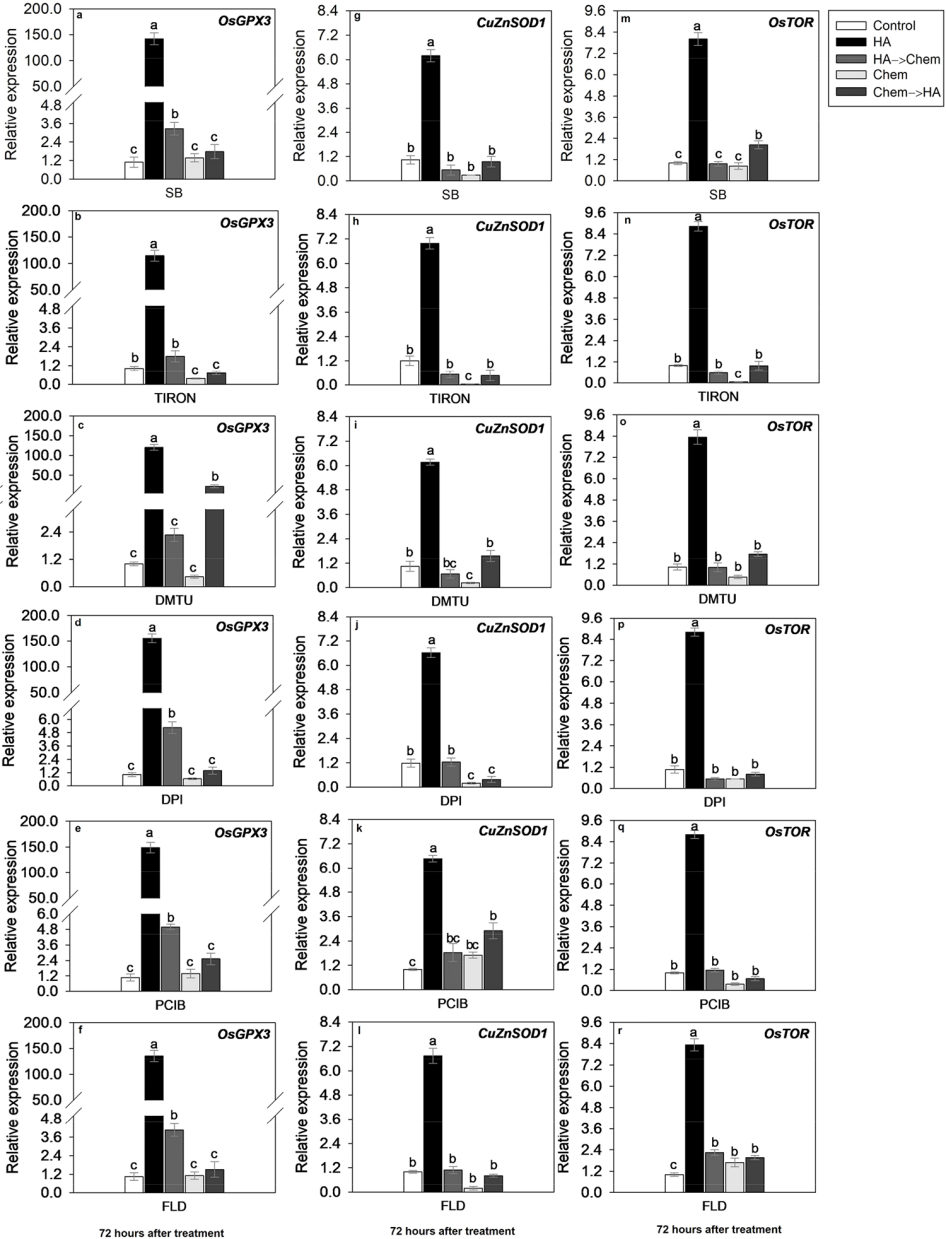
Root development involves an increase in expression level of *OsGPX3* (glutathione peroxidase), *CuZnSOD1* ([Cu–Zn] superoxide dismutase 1) and *OsTOR* (Target of Rapamycin) genes, involved in ROS and hormone signaling pathways. Rice plants grown for 72 h in nutrient solution with HA and addition of scavenger or inhibitor. SB: sodium benzoate, HO* scavenger (HO*: hydroxyl radical), TIRON: 4,5-dihydroxy-1,3-benzene disulfonic acid. O_2_^•–^ scavenger (O_2_^•–^: superoxide anion). DMTU: *N*,*N*′-dimethylthiourea. H_2_O_2_ scavenger (H_2_O_2_: hydrogen peroxide). DPI: diphenyleneiodonium chloride. NADPH oxidase inhibitor (NADPH: nicotinamide adenine dinucleotide phosphate). PCIB: 2-(*p*-chlorophenoxy)-2-methylpropionic acid, auxin action inhibitor. FLD: fluridone. ABA biosynthesis inhibitor (ABA: abscisic acid). Chem: a chemical substance added to solution as an inhibitor or scavenger. Bars indicate the four biological replicates standard error. Different letters above the bars represent significant differences according to Tukey’s test (*p*-value ≤ 0.05).

The expression of the *OsGPX3* gene was significantly upregulated in the roots of HA-treated seedlings, reaching approximately 150-fold relative expression compared to the control (Figure 8a–f). In contrast, control plants showed basal expression levels of around 1.2. In treatments with ROS scavengers and hormone inhibitors alone, the level of expression of *OsGPX3* remained low (ranging from 1.0 to 1.2), similar to control levels. However, when HA was applied before the chemical treatments (HA→Chem), expression levels were notably reduced compared to those of HA alone, ranging from approximately 2.4 to 6.0 depending on the treatment, indicating partial suppression of the HA-induced gene response. Conversely, in treatments where plants were first exposed to scavengers/inhibitors and then transferred to HA solution (Chem→HA), *OsGPX3* expression remained relatively low (ranging from 1.0 to 1.2), reinforcing that the residual effect of some inhibitors, particularly DMTU, DPI, PCIB, and FLD, can limit HA-mediated stimulation. GPX3 is a mitochondrial isoform of H_2_O_2_-induced glutathione peroxidase, essential for H_2_O_2_ homeostasis and root and shoot development in rice.^41^ In addition, it has been shown that *OsGPX3* is involved in ABA response in rice plants.^42^ These findings confirm that *OsGPX3* is highly responsive to HA treatment and is modulated by oxidative and hormonal signaling pathways.

The expression of *CuZnSOD1* was significantly enhanced in HA-treated seedlings, with levels reaching approximately 7.2-fold compared to the control (Figure 8g–l). Control seedlings exhibited low expression (∼1.2), and treatments with SB, TIRON, DMTU, DPI, PCIB, or FLD alone maintained similarly low expression levels. When HA was applied prior to the application of scavengers or inhibitors (HA→Chem), *CuZnSOD1* expression was partially reduced, ranging from 1.0 to 2.4 depending on the chemical, suggesting that HA-induced expression can be suppressed by subsequent chemical treatments. In Chem→HA treatments, *CuZnSOD1* levels remained low (∼1.0–2.5), highlighting the persistent inhibitory effects of ROS scavengers (notably DMTU and DPI) and hormone-related inhibitors. These results indicate that HA stimulates *CuZnSOD1* expression as part of a redox signaling response, which is dependent on the presence of active ROS and hormone signaling components.

The expression of *BAS1* was significantly up-regulated in roots of HA-treated seedlings compared to the control seedlings (Figure S11). In addition, the results showed that the gene expression of *BAS1* is similar in all treatments to that of ROS scavengers. However, in the treatments which HA is applied before or after the scavenger/inhibitor application, the expression levels was similar to the control.

The *OsPRX112* gene (encodes to *peroxidase isoform 2)*, probably located in the apoplast (UniProt–Q0D3N0) and involved in H_2_O_2_ catabolism was significantly increased in roots of HA-treated seedlings compared to the control seedling (Figure S12). It is crucial to highlight that *OsPRX112* gene was strongly suppressed in DMTU-treated plants, an H_2_O_2_ scavenger, however when the plants were transferred from DMTU to AH the expression level of *OsPRX112* was higher than control treatment (Figure S12c), the same observed in *OsGPX3* gene (Figure 8g). These results showed that despite the residual effect of DMTU already demonstrated (Figure 2c), the application of HA was still able to alter the oxidative metabolism—either by directly inducing changes or because the increase in H_2_O_2_ levels caused by HA overcame the residual scavenging effect of DMTU.

In addition, *OsPRX112* gene expression was also strongly suppressed with the application of DPI, PCIB and FLD, indicating a crosstalk between ROS, auxin and ABA (Figure S12d–f, respectively). This crosstalk between ROS and hormonal signaling pathways is well reported during stressful events.^43^

In addition to the results of shoot and root dry weights and root morphology, HA-treated rice plants showed a significant increase in the expression of Os*TOR* (*Target of Rapamycin*) (Figure 8m–r), which is essential for auxin signal transduction and promoting ABA biosynthesis, and Os*IAA11* (*3-Indolacetic acid 11*) (Figure S13), which is directly linked to auxin signaling pathways.

The expression of *OsTOR*, a central regulator of growth and hormonal signaling,^44^ was significantly upregulated in roots of HA-treated plants, reaching approximately 8.5-fold compared to the control (Figure 8m–r). In control seedlings, expression remained at the basal level (∼1.2). Treatments with SB, TIRON, DMTU, DPI, PCIB, and FLD alone led to strong downregulation, with expression levels between 1.0 and 2.5. In the HA→Chem treatments, the expression decreased substantially (ranging from 1.0 to 2.5), indicating suppression of HA effects by chemical inhibitors. Notably, in Chem→HA treatments, *OsTOR* expression did not recover significantly and remained similar to control levels (∼1.2), reinforcing the idea that these inhibitors, especially TIRON, DMTU, DPI and PCIB, exhibit a lasting suppressive effect on HA-induced gene activation. These findings support the hypothesis that *OsTOR* expression is regulated by both ROS and auxin/ABA signaling and that HA activates this gene as part of a broader growth-promoting molecular mechanism.

Furthermore, the root *OsIAA11* gene expression was increased in HA-treated plants, on the other hand, O_2_^•–^ scavenger, H_2_O_2_ scavenger, NADPH oxidase inhibitor, inhibitor of the auxin action and inhibitor of ABA biosynthesis application decreased the *OsIAA11* gene expression (Figure S13b–f, respectively).

## Discussion

The results of this study support the hypothesis that humic acid (HA) extracted from vermicompost enhances rice root growth through a complex mechanism involving redox signaling and hormonal regulation. Rather than merely reporting growth stimulation, we aimed to clarify the molecular and physiological basis of this response by integrating gene expression data and approaches with ROS scavengers and hormone inhibitors.

Upon HA application, increased levels of reactive oxygen species (ROS), such as H_2_O_2_ and O_2_^•–^ were detected in the roots. These ROS, typically associated with cellular stress, act in this context as signaling molecules that regulate developmental processes.^45^ The inhibition of NADPH oxidase activity or the scavenging of ROS significantly reduced HA-induced root proliferation, indicating that ROS accumulation is not a secondary consequence but rather a key trigger of the observed developmental responses. NADPH oxidase appears to play a central role in this process, as its inhibition diminished both ROS levels and root architectural changes.

Beyond redox signaling, gene expression analyses showed that HA modulates the transcription of several genes involved in hormone signaling pathways, such as *OsIAA11* and *OsTOR*. The suppression of HA-induced root growth by auxin and ABA inhibitors, along with the downregulation of these genes under these conditions, reveals a crosstalk between ROS and hormonal pathways. This interaction likely amplifies the effects of HA on root development. The more sustained suppression caused by the ABA biosynthesis inhibitor FLD, compared to the auxin inhibitor PCIB, suggests a more persistent role for ABA in maintaining HA-mediated responses.

Also, our gene expression analysis revealed that HA significantly upregulated genes associated with antioxidant enzymes (*OsGPX3*, *CuZnSOD1*, *BAS1*, *OsPRX112*, Figures 8, S11 and S12), calcium signaling (*OsCPK7*, *OsTPC1*, Figures S14 and S15, respectively), proton pumping (*OsA7*, Figure S16) and vesicle trafficking (*TOM1*, *SEC1B*, Figures S17 and S18, respectively). These genes are known to play roles in root architecture and nutrient uptake^46–49^ and their expression patterns were strongly altered by treatments that interfered with ROS or hormonal pathways.

These findings align with studies in maize, where humic substances extracted from vermicompost promoted root growth via auxin-related signaling pathways influenced by hormone inhibitors such as TIBA and PCIB.^50^ Furthermore, it was demonstrated that humic acids modify root architecture in *Arabidopsis* through H^+^-ATPase-dependent target of rapamycin (*TOR*) activation in concert with Ca^2+^ and ROS signaling, highlighting an integrated regulatory network that modulates root development.^51^ Complementarily, it was showed that humic substances affect *Arabidopsis* physiology by altering the expression of genes involved in primary metabolism, growth, and development, highlighting the broad impact of HA on plant gene regulation.^52^

HA treatment also upregulated *OsNRT2.1*, a nitrate transporter gene responsive to ABA and critical for nitrogen uptake and root development.^53^ The downregulation of this gene under ABA inhibition (Figure S21f) reinforces the involvement of a ROS–ABA signaling pathways in HA-induced responses. Additionally, increased expression of vesicle trafficking genes (*TOM1*, *SEC1B,* Figures S17 and S18, respectively) and calcium signaling components (*OsCPK7*, *OsTPC1,* Figures S14 and S15, respectively) indicate a systemic cellular response involving intracellular transport and secondary messengers, further supporting a broad reprogramming of root physiology.

Interestingly, genes involved in primary metabolism (*HXK5*) and cytoskeletal regulation (*PHS1*)^52^ were also induced (Figures S19 and S20, respectively), suggesting that HA influences cellular energy dynamics and microtubule stability, factors directly related to root elongation and tip expansion. These findings complement previous transcriptomic studies^54,55^ and provide mechanistic insights into how HA modulates root system efficiency and adaptability.

It is important to consider that humic acids are chemically heterogeneous, with a wide range of functional groups capable of interacting with both organic and inorganic molecules. The simultaneous application of HA and chemical inhibitors or scavengers may lead to partial complexation or adsorption, affecting the inhibitor availability and efficacy. To account for this, we performed a time-course experiment to assess the duration of inhibitor activity postnutrient renewal. This allowed us to differentiate between true physiological effects and potential chemical interference.

Overall, these findings support a model in which HA acts as a priming agent, initiating ROS accumulation that integrates with hormonal and metabolic signaling networks. This coordinated response promotes adaptive root development, enhancing the plant’s capacity for nutrient and water uptake. Although some aspects of HA’s complex interaction with plant systems remain to be fully elucidated, our study provides valuable insights into the molecular basis of its biostimulant effects in rice.

### Practical and Environmental Implications

From an environmental research perspective, understanding how humic substances trigger plant growth through endogenous signaling mechanisms opens possibilities for sustainable agriculture. Unlike synthetic growth promoters or chemical fertilizers, HA acts by enhancing plant regulatory systems. The ability of HA to stimulate key pathways involved in nutrient uptake, stress tolerance, and root system development could improve crop productivity under reduced fertilizer inputs or adverse growing conditions, a key goal in the face of climate change and environmental degradation.

These findings also suggest new biomarkers (e.g., *OsCPK7*, *OsTPC1*, and *TOM1*) for evaluating HA effectiveness in different crop genotypes or soil conditions, which could inform the development of next-generation biostimulants. Moreover, given that ROS are typically associated with abiotic stress responses, employing their controlled accumulation as a positive signal through HA treatment presents a novel approach to increasing plant resilience.

Although HA was extracted using the IHSS protocol, commonly adopted for scientific purposes due to its reproducibility and capacity to isolate defined chemical fractions, we recognize that this method employs strong alkaline reagents and does not fully align with green chemistry principles. From a practical standpoint, the direct use of vermicompost or its water-soluble organic compounds may offer more sustainable alternatives. Vermicompost is already rich in bioactive molecules, nutrients, and beneficial microorganisms, making it effective as a soil amendment, even without purification steps.

The purpose behind using purified HA in this study was to allow mechanistic exploration of humic acid bioactivity under controlled conditions, separating its effects from those of the complex composition of vermicompost. Nonetheless, future research should prioritize greener extraction methods or field validation of crude compost extracts to ensure their environmental and economic viability.

Further research should explore the interaction between HA and plants under field conditions, where multiple environmental factors occur simultaneously. This will help to bridge the gap between controlled studies and practical agricultural applications. In our study, HA application enhanced root development and activated pathways related to nutrient uptake and stress signaling, indicating greater potential for plants to access and uptake nutrients efficiently. Such improvements in root architecture and signaling may allow crops to maintain productivity under low nutrient availability or stress conditions. By improving nutrient use efficiency (NUE), humic acids could reduce the dependency on high fertilizer inputs, a key step toward more sustainable crop management. Therefore, our findings offer a mechanistic basis to support the claim that HA applications may contribute to reduced fertilizer use and, consequently, to decreased environmental degradation associated with excessive nutrient input into agricultural systems.

## Supplementary Material



## Notes

FTIR spectra of humic acid (HA) from vermicompost; ^13^C–CP/MAS NMR spectra of humic acid (HA) from vermicompost; integration of ^13^C–CP/MAS NMR spectra area of carbons regions of humic acid (HA); dose–response curve of HA concentrations; dose–response curve for: SB concentrations (HO* scavenger), TIRON concentrations (O_2_^•–^ scavenger), DMTU concentrations (H_2_O_2_ scavenger), DPI concentrations (NADPH oxidase inhibitor), PCIB concentrations (auxin action inhibitor), FLD concentrations (ABA biosynthesis inhibitor); primers sequences used in the RT-PCR; effect of HA, SB, TIRON, DMTU, DPI, PCIB, and FLD on shoot and root development; gene expression profiles related to root development and signaling pathways (ROS, hormone, calcium, vesicle transport, metabolism, and nitrogen uptake) (PDF)

## Abbreviations

ABA: abscisic acid
NH_4_^+^: ammonium
GUS: β-glucuronidase
DPI: diphenyleneiodonium chloride
FLD: fluridone
HA: humic acid
HS: humic substances
H_2_O_2_: hydrogen peroxide
HO*: hydroxyl radical
IS: ionic strength
NADPH: nicotinamide adenine dinucleotide phosphate
DMTU: *N*,*N*′-dimethylthiourea
NO_3_^–^: nitrate
ROS: reactive oxygen species
SB: sodium benzoate
O_2_^•–^: superoxide anion
PCIB: 2-(*p*-chlorophenoxy)-2-methylpropionic acid
TIRON: 4,5-dihydroxy-1,3-benzene disulfonic acid.
