# Supplementary material for: Humic Acid Regulates Root Growth through ROS-Dependent Pathway and Hormone Signaling in Rice
Source: J Agric Food Chem. 2025 Jul 31;73(32):20081–93. doi: 10.1021/acs.jafc.5c06288 (PMC12355950; doi:10.1021/acs.jafc.5c06288)
Supplement: Supplementary file 1 [file jf5c06288_si_001.pdf]

Supporting Information

Humic acid regulates root growth through ROS-dependent pathway and hormone signalling in rice

Andressa Fabiane Faria de Souza[1] • Andrés Calderín García[2] • José Nivaldo de Oliveira Sátiro[1] • Brisa Ribeiro de Lima[1] • Manlio Silvestre Fernandes[1], • Ricardo Luiz Louro Berbara[2] • Leandro Azevedo Santos[1*]

Affiliations

[1]Plant Nutrition Laboratory, Department of Soils, Federal Rural University of Rio de Janeiro (UFRRJ), Seropédica 23890-000, RJ, Brazil

[2]Soil Biological Chemistry Laboratory, Department of Soils, Federal Rural University of Rio de Janeiro (UFRRJ), Seropédica 23890-000, RJ, Brazil

Corresponding author

Correspondence to Leandro Azevedo Santos: azevedo.ufrrj@gmail.com

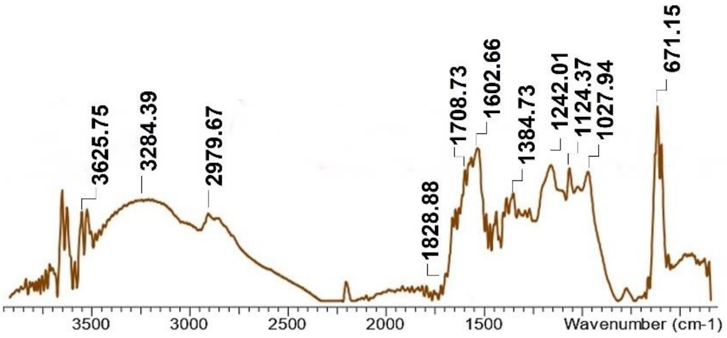

Figure S1. FTIR spectra of humic acid (HA) from vermicompost.

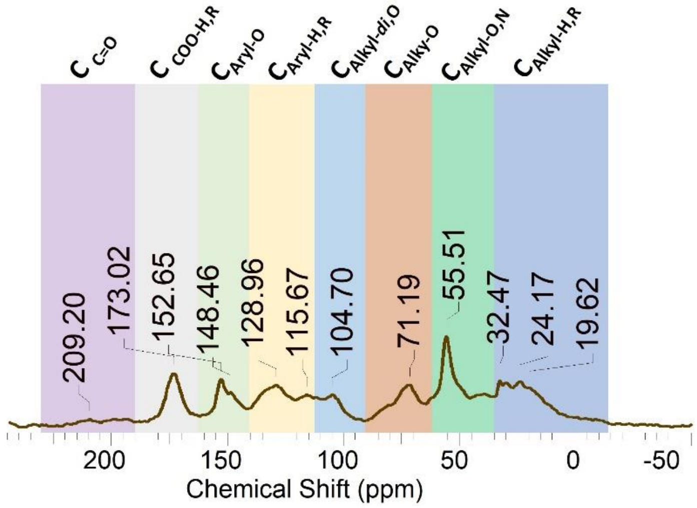

Figure S2. ^{13}C-CP/MAS-NMR spectra of humic acid (HA) from vermicompost.

Table S1. Integration of ^{13}C-CP/MAS-NMR spectra area of carbons regions of humic acid (HA) from vermicompost

	C_{Alkyl}-H,R	C_{Alkyl}-O,N	C_{Alky}-O	C_{Alkyl}-*di*-O	C_{Aryl}-H,R	C_{Aryl}-O	C_{COO}-H,R	$C_{C=O}$
	**0-45 ppm**	**45-60 ppm**	**60-90 ppm**	**90-110 ppm**	**110-140 ppm**	**140-155 ppm**	**155-185 ppm**	**185-230 ppm**
	--------------------	--------------------	--------------------	--------------------%	--------------------	--------------------	--------------------	--------------------
**HA**	31.30	12.60	13.10	6.10	13.10	6.10	13.10	4.50
	**Aro**	**Ali**	**HB/HI**	**P**				
	19.22	80.78	1.02	1.25				

Aro = aromaticity (%); Ali = aliphaticity (%); HB/HI = hydrophobicity index; P = polarity index.

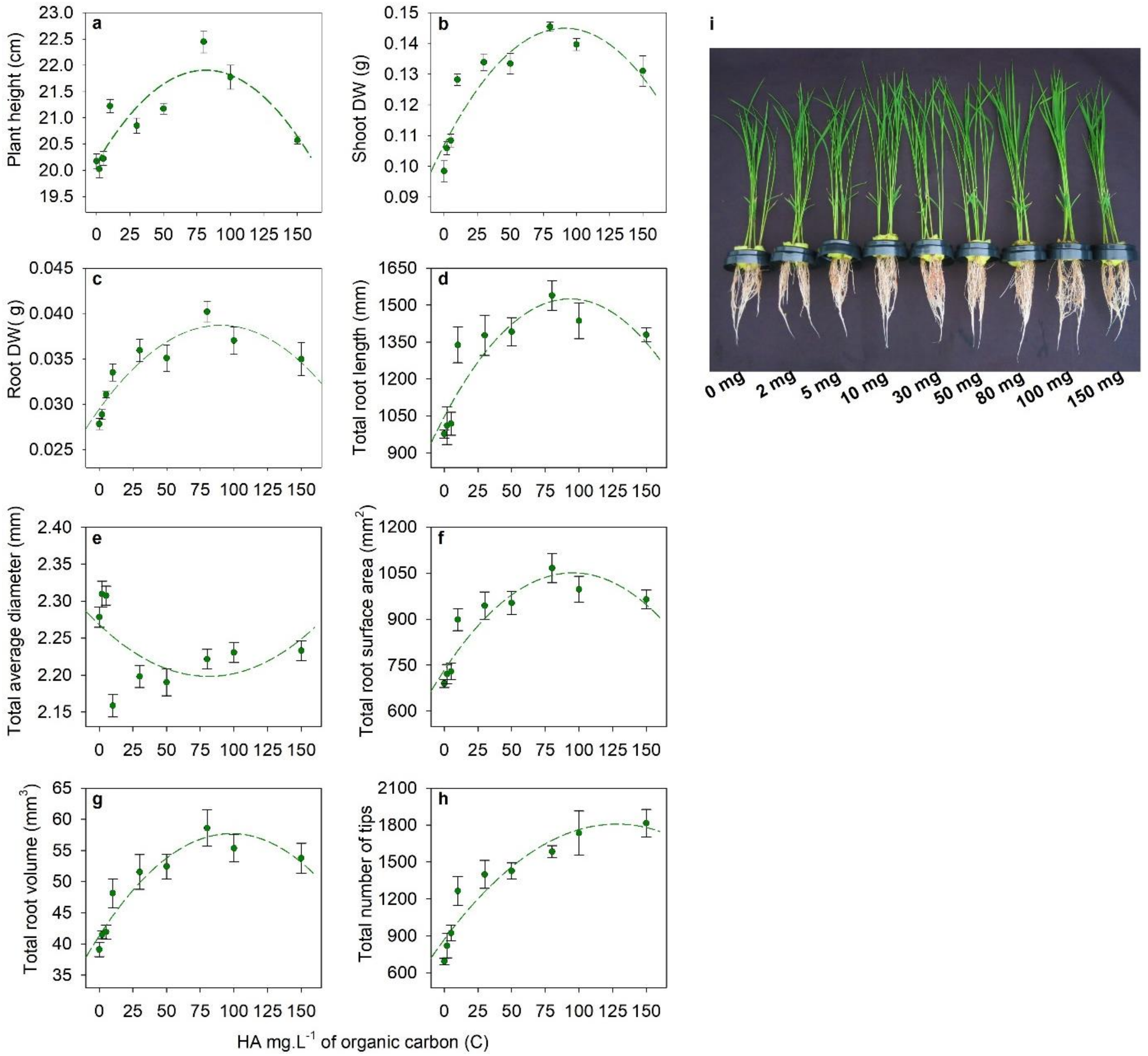

Figure S3. Dose-response curve of HA concentrations. The plants were cultivated in a 50 mL Falcon tube, two applications of HA were applied, at 6 DAG and 9 DAG, and were harvested at 12 DAG. (a-c) Physiological measurement and shoot and root biomass of rice plants during exposure to HA. (d-h). Root morphology of rice plants during exposure to HA. The analysis was carried out using WinRhizo Arabidopsis software. (i) Plants grown in different concentrations of HA. HA: humic acid, DAG: days after germination, DW (g): dry weight in grams. Concentrations evaluated: 0 (control plants), 2, 5, 10, 30, 50, 80, 100 and 150 mg.L^{-1} of organic carbon (C). Bars indicate the four biological replicates standard error. Quadratic regression was fitted.

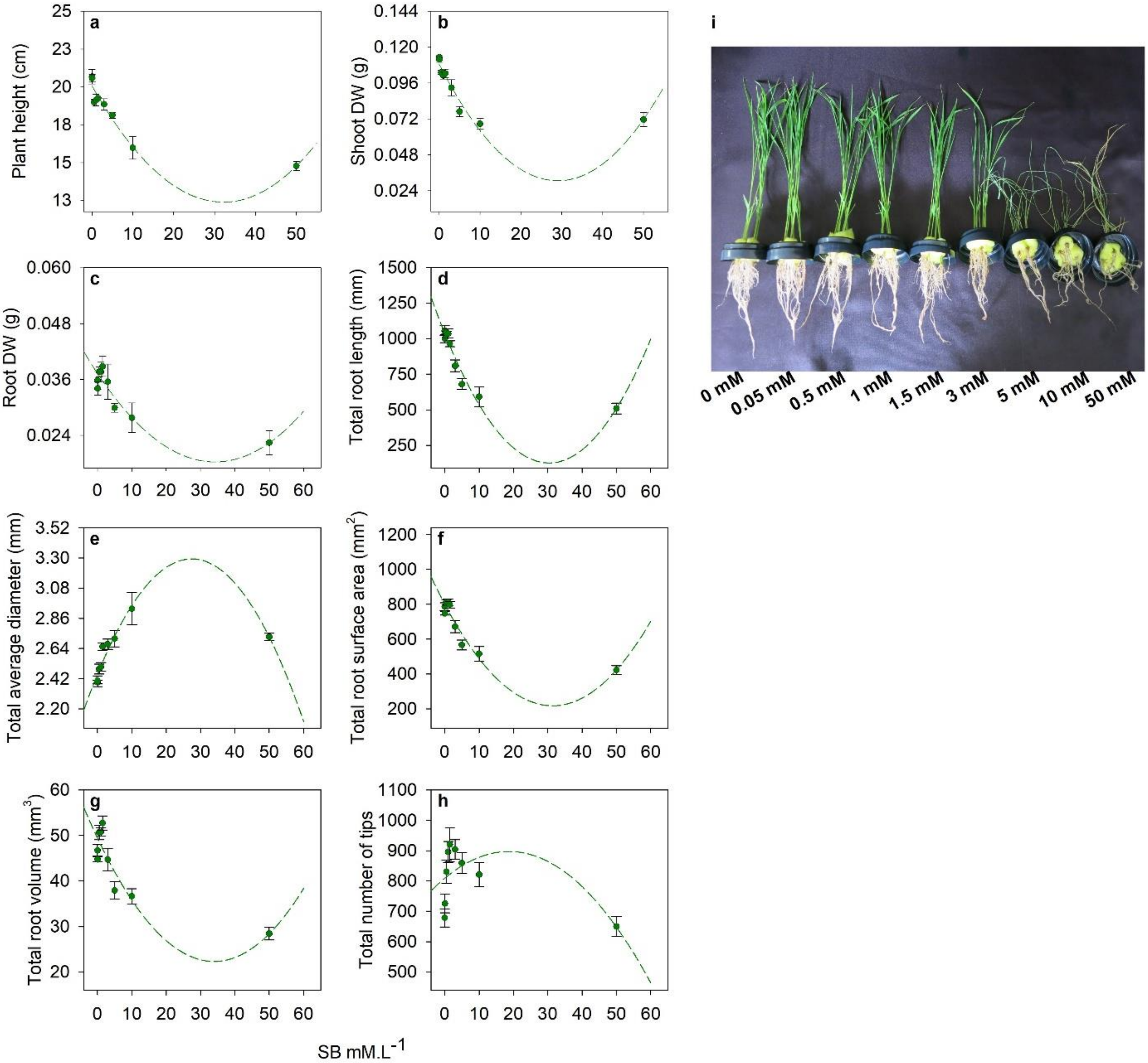

Figure S4. Dose-response curve of SB concentrations, HO* scavenger. The plants were harvested at 12 DAG and one application of SB was applied at 9 DAG. (a-c) Physiological measurement and shoot and root biomass of rice plants during exposure to SB. (d-h). Root morphology of rice plants during exposure to SB. The analysis was carried out using WinRhizo Arabidopsis software. (i) Plants grown in different concentrations of SB. SB: sodium benzoate, HO*: hydroxyl radical, DAG: days after germination, DW (g): dry weight in grams. Concentrations evaluated: 0 (control plants); 0.05; 0.5; 1; 1.5; 3; 5; 10 and 50 mM L^{-1} of SB. Bars indicate the four biological replicates standard error. Quadratic regression was fitted.

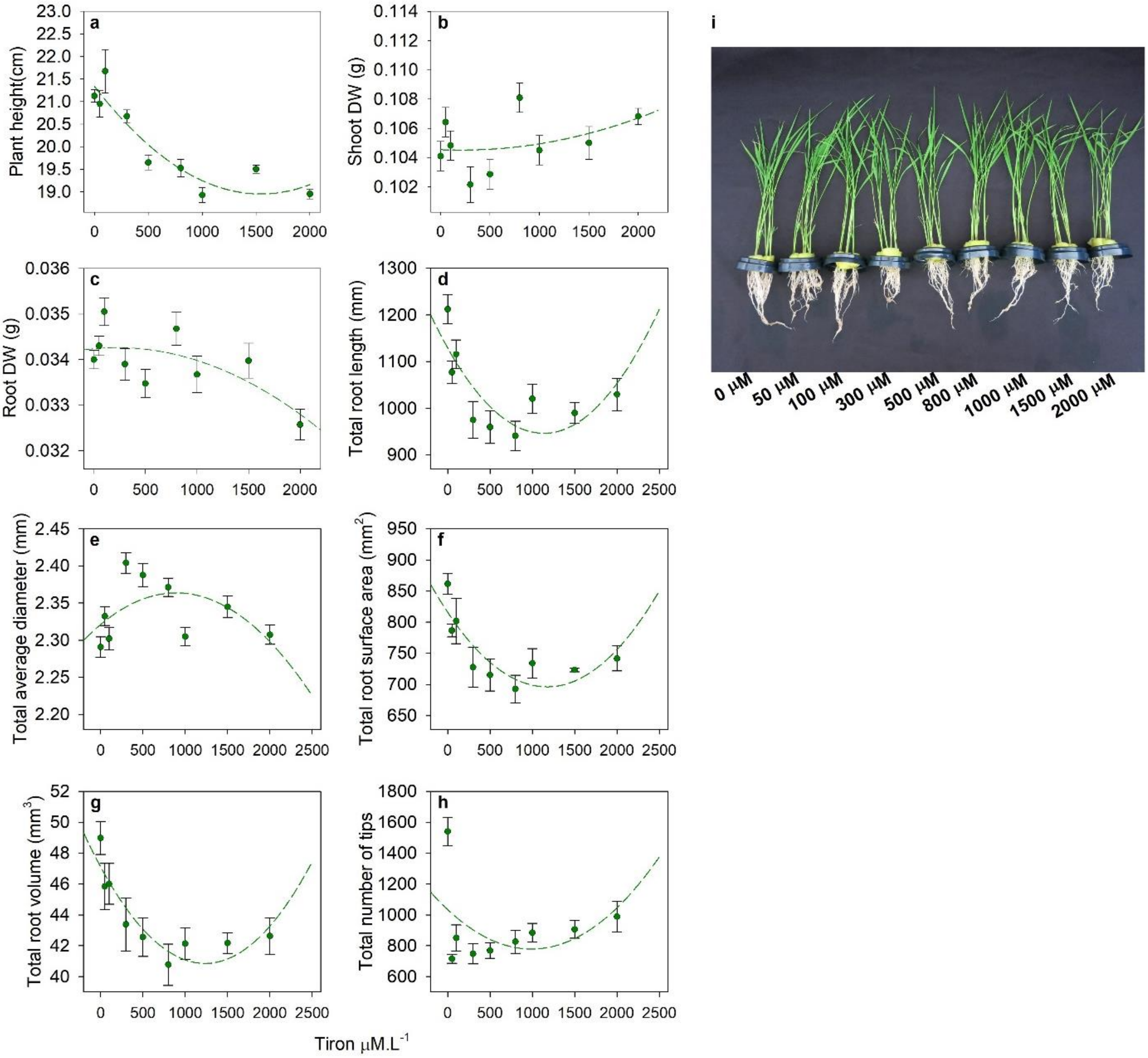

Figure S5. Dose-response curve of TIRON concentrations, $O_2^{\bullet-}$ scavenger. The plants were harvested at 12 DAG and one application of TIRON was applied at 9 DAG. (a-c) Physiological measurement and shoot and root biomass of rice plants during exposure to TIRON. (d-h). Root morphology of rice plants during exposure to TIRON. The analysis was carried out using WinRhizo Arabidopsis software. (i) Plants grown in different concentrations of TIRON. TIRON: 4,5-dihydroxy-1,3-benzene disulfonic acid, $O_2^{\bullet-}$: superoxide anion, DAG: days after germination, DW (g): dry weight in grams. Concentrations evaluated: 0 (control plants); 50; 100; 300; 500; 800; 1000; 1500 and 2000 µM L^{-1} of TIRON. Bars indicate the four biological replicates standard error. Quadratic regression was fitted.

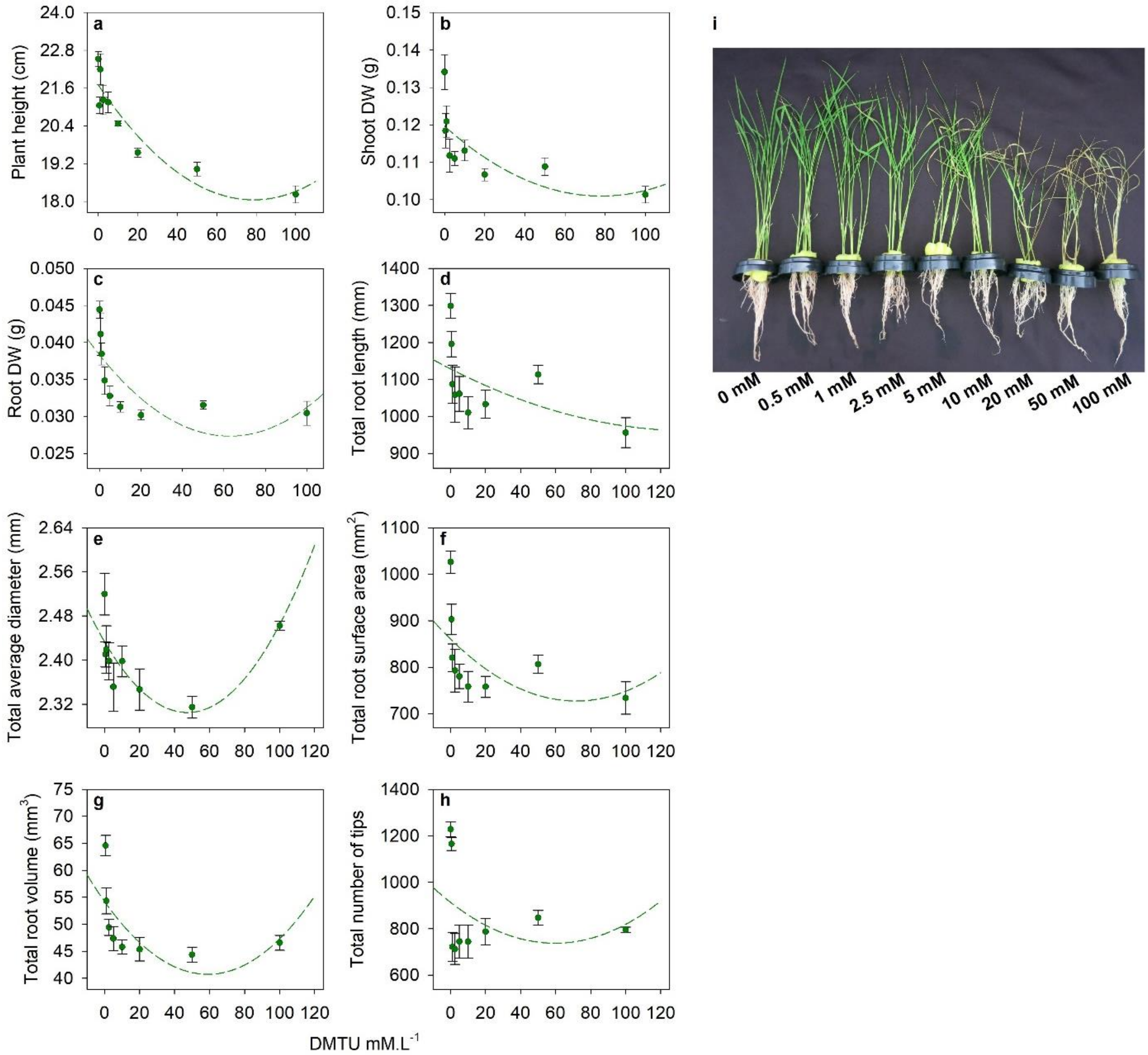

Figure S6. Dose-response curve of DMTU concentrations, H_2O_2 scavenger. The plants were harvested at 12 DAG and one application of DMTU was applied at 9 DAG. (a-c) Physiological measurement and shoot and root biomass of rice plants during exposure to DMTU. (d-h). Root morphology of rice plants during exposure to DMTU. The analysis was carried out using WinRhizo Arabidopsis software. (i) Plants grown in different concentrations of DMTU. DMTU: N N'-Dimethylthiourea, H_2O_2: hydrogen peroxide, DAG: days after germination, DW (g): dry weight in grams. Concentrations evaluated: 0 (control plants); 0.5; 1; 2.5; 5; 10; 20, 50 and 100 mM L^{-1} of DMTU. Bars indicate the four biological replicates standard error. Quadratic regression was fitted.

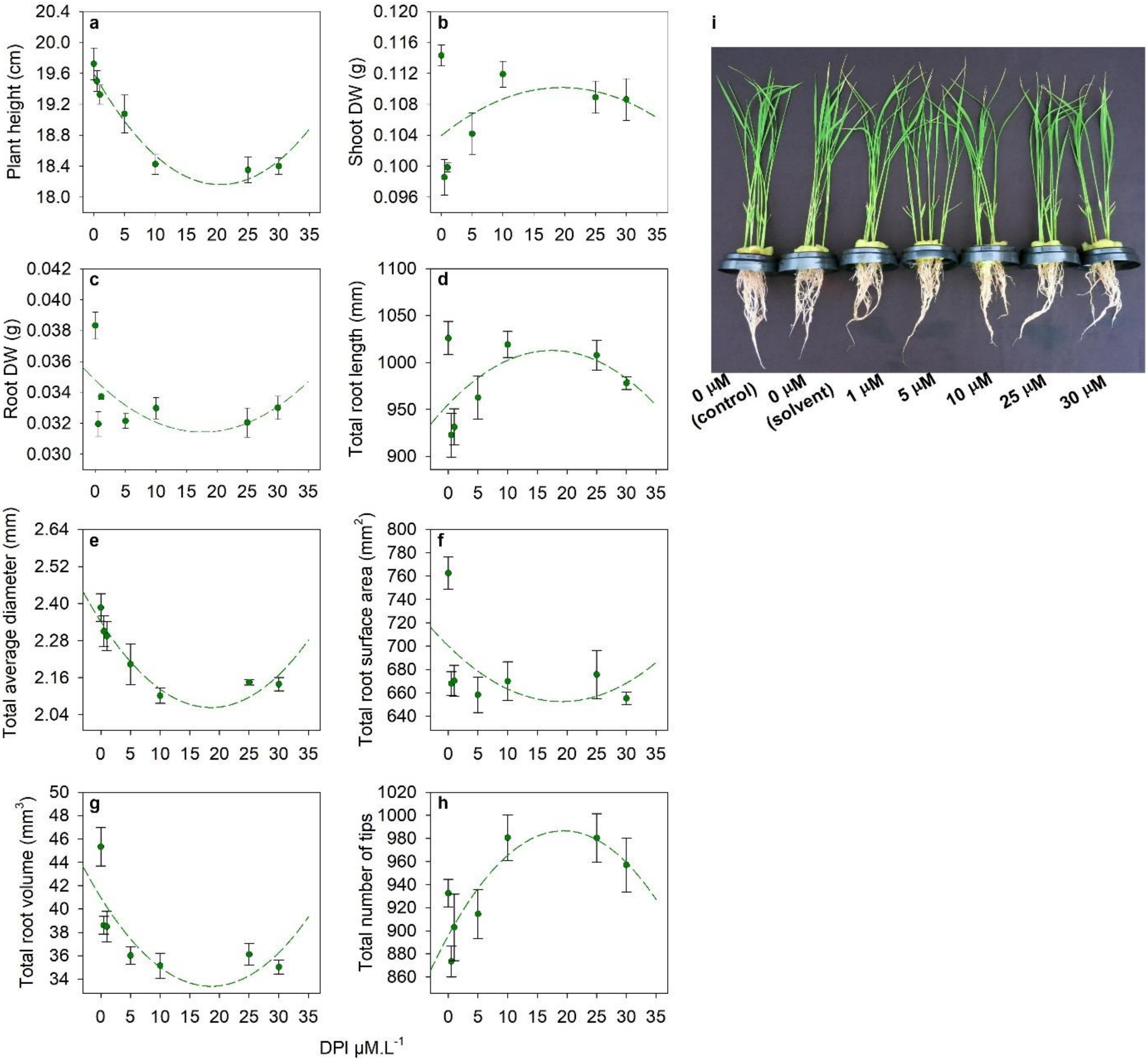

Figure S7. Dose-response curve of DPI concentrations, NADPH oxidase inhibitor. The plants were harvested at 12 DAG and one application of DPI was applied at 9 DAG. (a-c) Physiological measurement and shoot and root biomass of rice plants during exposure to DPI. (d-h). Root morphology of rice plants during exposure to DPI. The analysis was carried out using WinRhizo Arabidopsis software. (i) Plants grown in different concentrations of DPI. DPI: Diphenyleneiodonium chloride, NADPH: nicotinamide adenine dinucleotide phosphate, DAG: days after germination, DW (g): dry weight in grams. Concentrations evaluated: 0 (control plants); 0 (control plants with solvent); 1; 5; 10; 25 and 30 µM L^{-1} of DPI. Bars indicate the four biological replicates standard error. Quadratic regression was fitted.

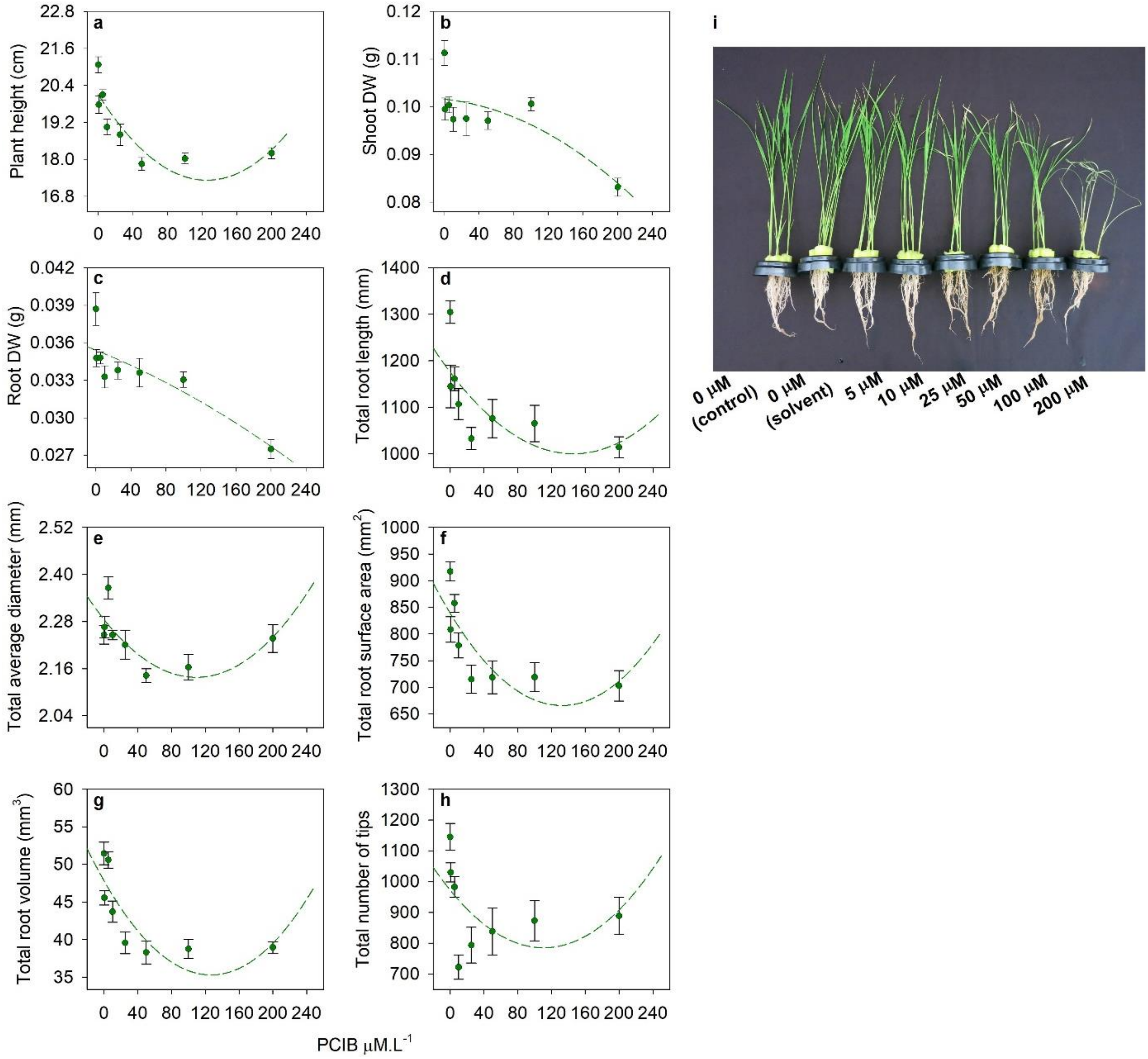

Figure S8. Dose-response curve of PCIB concentrations, auxin action inhibitor. The plants were harvested at 12 DAG and one application of PCIB was applied at 9 DAG. (a-c) Physiological measurement and shoot and root biomass of rice plants during exposure to PCIB. (d-h). Root morphology of rice plants during exposure to PCIB. The analysis was carried out using WinRhizo Arabidopsis software. (i) Plants grown in different concentrations of PCIB. PCIB: 2-(p-chlorophenoxy)-2-methylpropionic acid, DAG: days after germination, DW (g): dry weight in grams. Concentrations evaluated: 0 (control plants); 0 (control plants with solvent); 5; 10; 25; 50; 100 and 200 $\mu M\ L^{-1}$ of PCIB. Bars indicate the four biological replicates standard error. Quadratic regression was fitted.

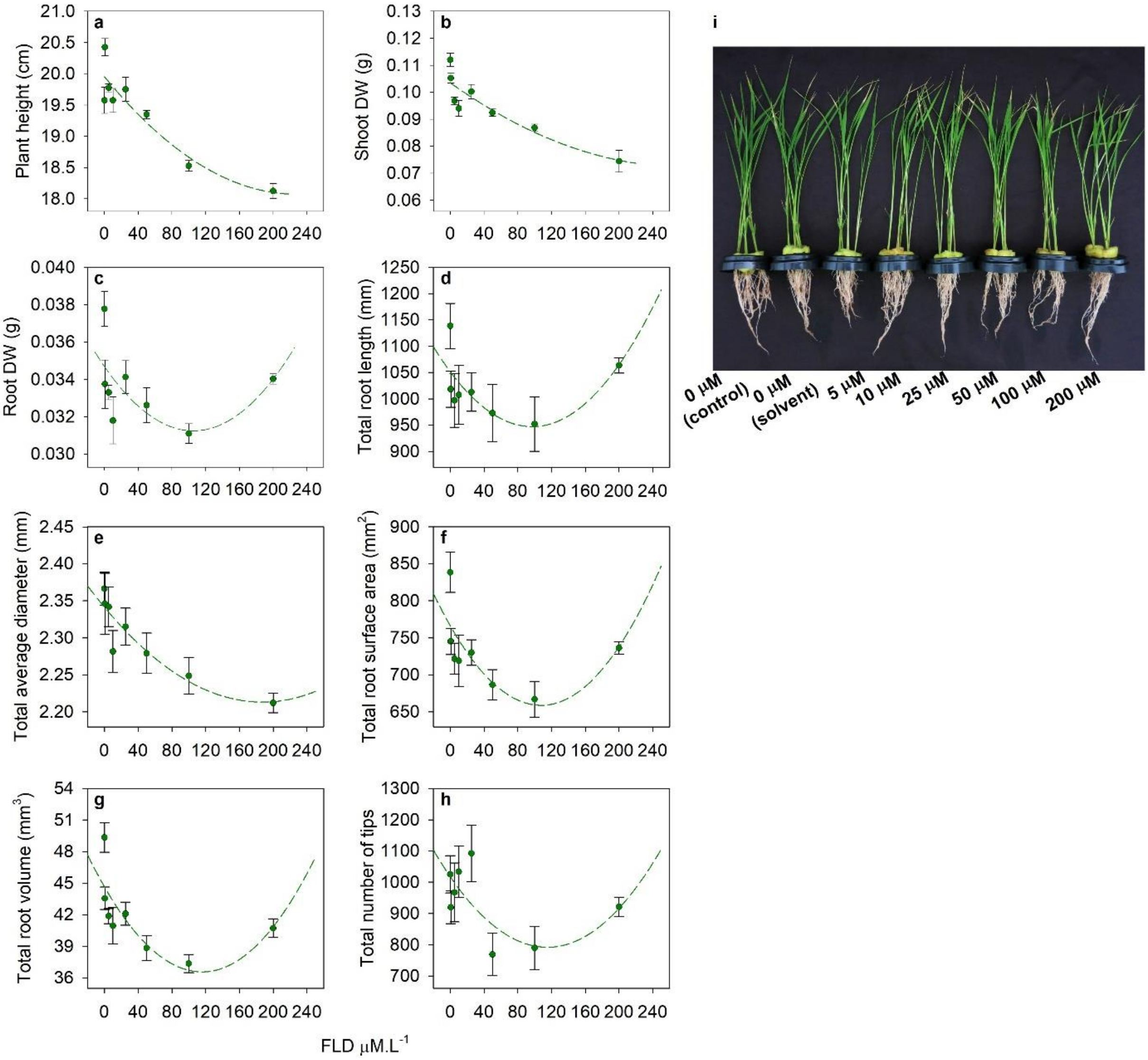

Figure S9. Dose-response curve of FLD concentrations, ABA biosynthesis inhibitor. The plants were harvested at 12 DAG and one application of FLD was applied at 9 DAG. (a-c) Physiological measurement and shoot and root biomass of rice plants during exposure to FLD. (d-h). Root morphology of rice plants during exposure to FLD. The analysis was carried out using WinRhizo Arabidopsis software. (i) Plants grown in different concentrations of FLD. FLD: Fluridone, ABA: abscisic acid, DAG: days after germination, DW (g): dry weight in grams. Concentrations evaluated: 0 (control plants); 0 (control plants with solvent); 5; 10; 25; 50; 100 e 200 µM L^{-1} of FLD. Bars indicate the four biological replicates standard error. Quadratic regression was fitted.

Table S2. Sequences of the forward and reverse primers used in the RT-qPCR reactions.

**Gene Name**	***Forward primer***	***Reverse primer***
OsUBQ5	5’-ACCACTTCGACCGCCACTACT-3’	5’-ACGCCTAAGCCTGCTGGTT-3’
BAS1	5’-GTCTGCCCGACCGAGATTAC-3’	5’-CCAAGCCCACCAGATTTCCT-3’
OsGPX3	5’-CTGCGTTGCATTGAGCACTT-3’	5’-GGGGCAAAGTGATGCAGTAAG-3’
OsPrx112	5’-ACATCTCGCCCTTGACTGGA-3’	5’-CAATGTCGGGGCCTAGCTTT-3’
CuZnSOD1	5’-AGATTCCAAACCAGCAGGAGT-3’	5’-AAGCACAACAACAGCCTTCAC-3’
PHS1	5’-TTACCAAGGCGTTCGCTGAT-3’	5’-ACATCACCACACTGCCACAA-3’
TOR	5’-GGCTGAATGGATGAGGCACT-3’	5’-CTCACGACCAACAGAAGGCT-3’
SEC1B	5’-CGAAGCTGAAGATGCTAACTGC-3’	5’-CGTAGCAATGGGTTTCTTACCAA-3’

TOM1	5'-TACAGCAACAGCCAAGGGAG-3'	5'-TGTAGAGTAGGGCGAGCTGT-3'
HXK5	5'-CAAGATGCCAAAAGACGGCA-3'	5'-CCACCGAAGACGAGACATCA-3'
IAA11	5'-AGCTGAGAGATGACCTGGAGT-3'	5'-AAGGCCAATGGCTTCAGACC-3'
OsA7	5'-TCCAACACGCTCTTCAACGA-3'	5'-CCTTCAGCTTCACCACCGAT-3'
OsNRT2.1	5'-CCACGGTGCAAGTCTCAAGT-3'	5'-GTCGCAGAATTGTTTACGCCT-3'
OsCPK7	5'-TAGCCCTCGAAGAAGCAAGG-3'	5'-TCCCCAACCTTACGAGACCT-3'
OsTPC1	5'-CGGAGCCCACTGTTTGAGTA-3'	5'-TCTTGCCACACTTTCTGCGA-3'

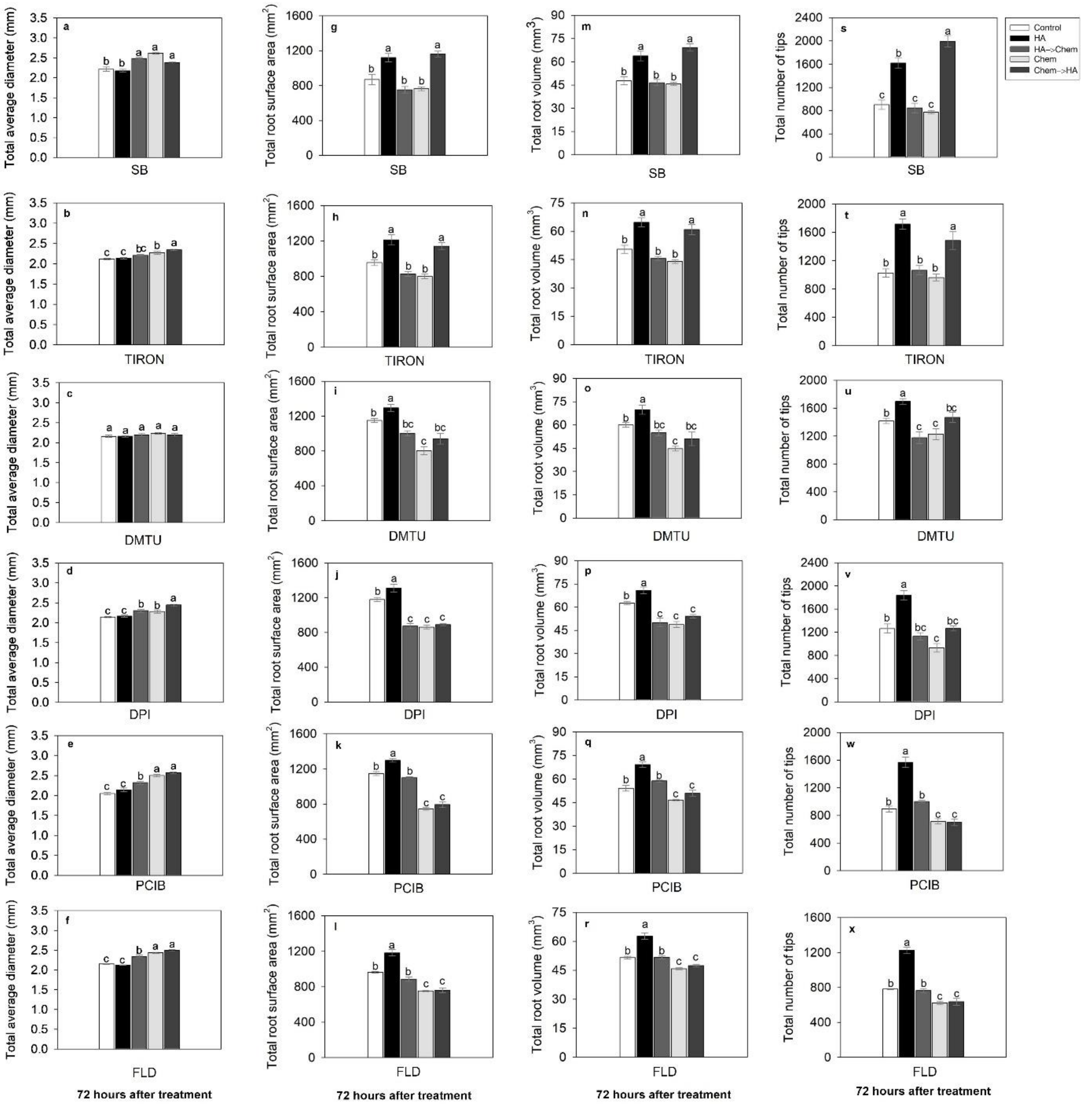

Figure S10. Effect of 80 mg.L^{-1} of organic carbon from HA, SB (HO* scavenger), and TIRON ($O_2^{\bullet -}$ scavenger), DMTU (H_2O_2 scavenger), and DPI (NADPH oxidase inhibitor), PCIB (auxin action inhibitor), and FLD (ABA biosynthesis inhibitor) on shoot and root development. Rice plants grown for 72 hours in nutrient solution with HA and addition of scavenger / inhibitor. (a-f) Total average diameter (mm). (g-l) Total root surface area (mm^2). (m-r) Total root volume (mm^3). (s-x) Total number of tips. The analysis of root morphology was carried out using WinRhizo Arabidopsis software. SB: sodium benzoate, HO*: hydroxyl radical, TIRON: 4,5-dihydroxy-1,3-benzene disulfonic acid, $O_2^{\bullet -}$: superoxide anion, DMTU: N N'-Dimethylthiourea, H_2O_2: hydrogen peroxide, DPI: Diphenyleneiodonium chloride, NADPH: nicotinamide adenine dinucleotide phosphate, PCIB: 2-(p-

chlorophenoxy)-2-methylpropionic acid, FLD: Fluridone, ABA: abscisic acid, HA: humic acid, Chem: a chemical substance added to solution as scavenger or an inhibitor. Bars indicate the four biological replicates standard error. Different letters above the bars represent significant differences according to Tukey's test (p-value ≤0.05).

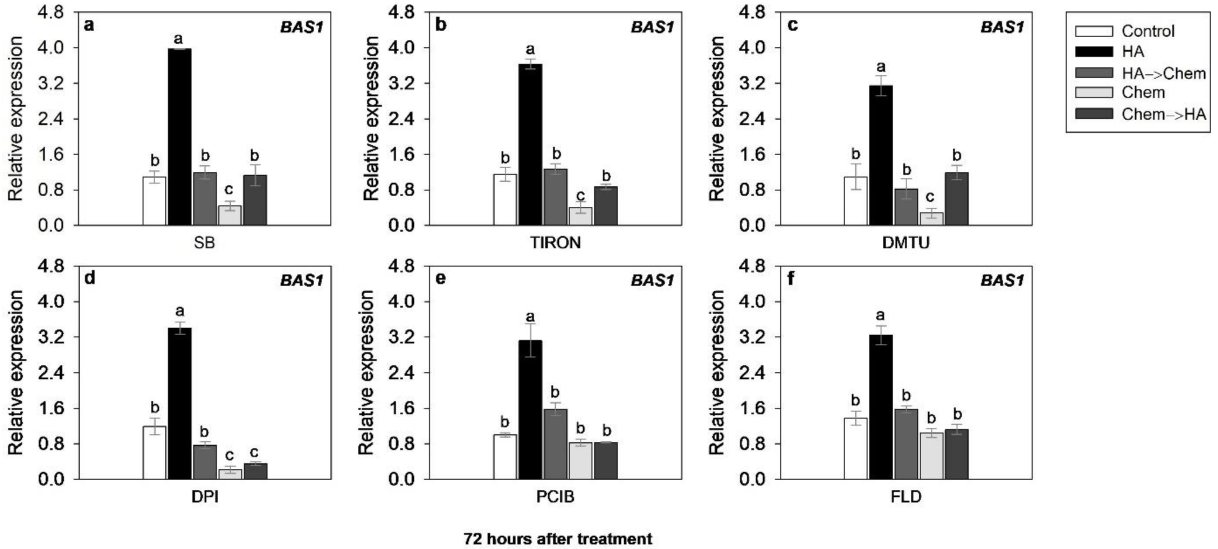

Figure S11. Root development involves an increase in expression level of *BAS1* gene, 2-Cys peroxiredoxin 1, involved in ROS signalling pathways. Rice plants grown for 72 hours in nutrient solution with HA and addition of scavenger or inhibitor (a) SB: sodium benzoate, HO* scavenger (HO*: hydroxyl radical). (b) TIRON: 4,5-dihydroxy-1,3-benzene disulfonic acid, $O_2^{\bullet-}$ scavenger ($O_2^{\bullet-}$: superoxide anion). (c) DMTU: N N'-Dimethylthiourea, H_2O_2 scavenger (H_2O_2: hydrogen peroxide). (d) DPI: Diphenyleneiodonium chloride, NADPH oxidase inhibitor (NADPH: nicotinamide adenine dinucleotide phosphate). (e) PCIB: 2-(p-chlorophenoxy)-2-methylpropionic acid, auxin action inhibitor. (f) FLD: Fluridone, ABA biosynthesis inhibitor (ABA: abscisic acid). Chem: a chemical substance added to solution as an inhibitor or scavenger. Bars indicate the four biological replicates standard error. Different letters above the bars represent significant differences according to Tukey's test (p-value ≤0.05).

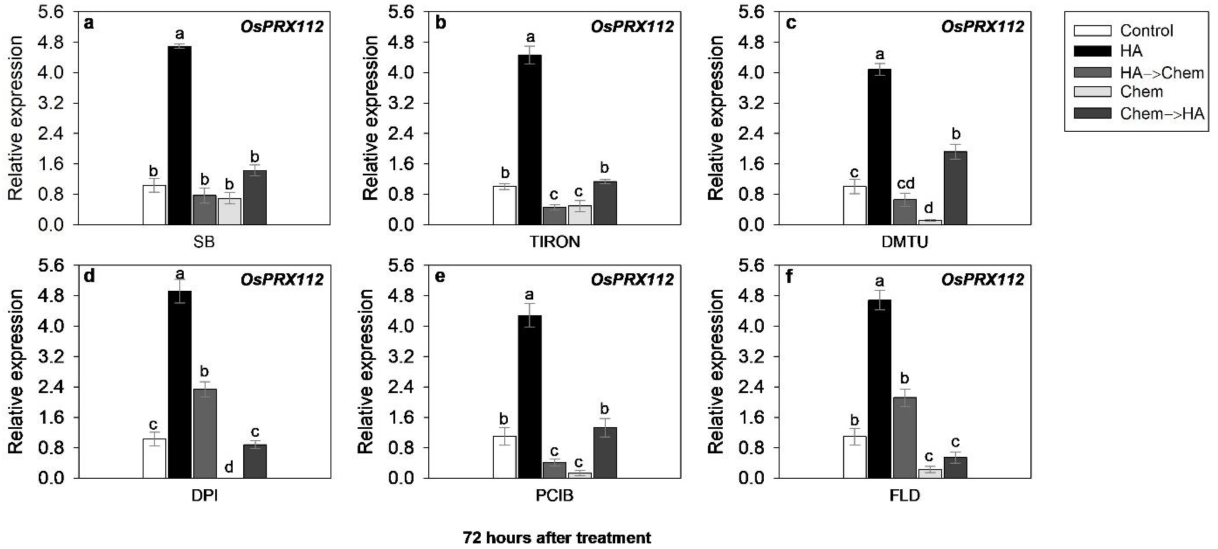

Figure S12. Root development involves an increase in expression level of *OsPRX112* gene, peroxidase isoform 2, involved in ROS signalling pathways. Rice plants grown for 72 hours in nutrient solution with HA and addition of scavenger or inhibitor (a) SB: sodium benzoate, HO* scavenger (HO*: hydroxyl radical). (b) TIRON: 4,5-dihydroxy-1,3-benzene disulfonic acid, $O_2^{\bullet-}$ scavenger ($O_2^{\bullet-}$: superoxide anion). (c) DMTU: N N'-Dimethylthiourea, H_2O_2 scavenger (H_2O_2: hydrogen peroxide). (d) DPI: Diphenyleneiodonium chloride, NADPH oxidase inhibitor (NADPH: nicotinamide adenine dinucleotide phosphate). (e) PCIB: 2-(p-chlorophenoxy)-2-methylpropionic acid, auxin action inhibitor. (f) FLD: Fluridone, ABA biosynthesis inhibitor (ABA: abscisic acid).

Chem: a chemical substance added to solution as an inhibitor or scavenger. Bars indicate the four biological replicates standard error. Different letters above the bars represent significant differences according to Tukey's test (p-value ≤0.05).

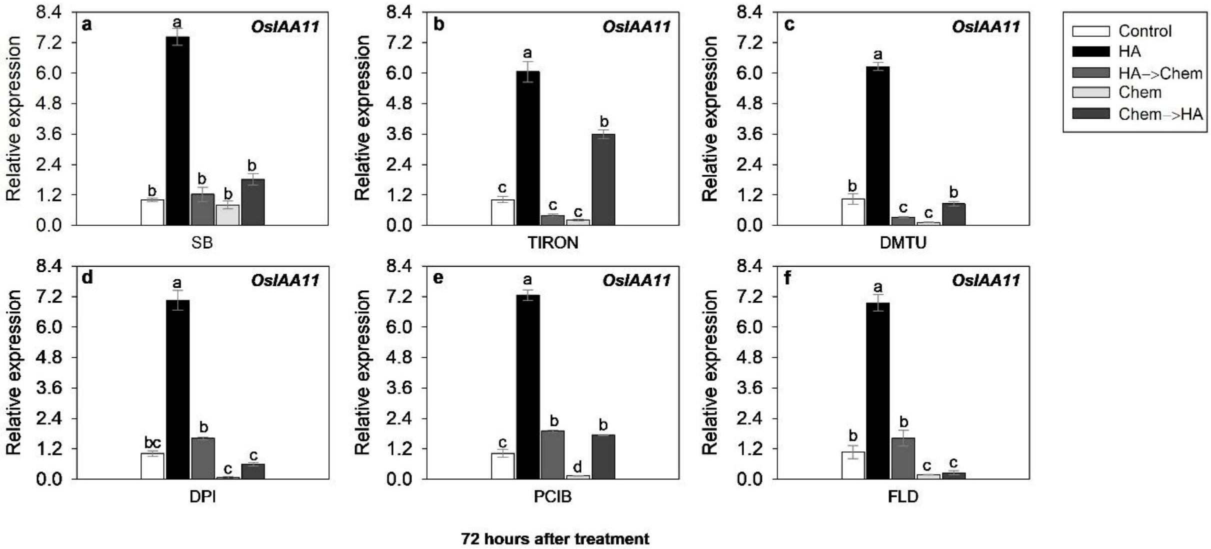

Figure S13. Root development involves an increase in expression level of *OsIAA11* gene, 3-Indolacetic acid 11, involved in hormonal signalling pathways. Rice plants grown for 72 hours in nutrient solution with HA and addition of scavenger or inhibitor (a) SB: sodium benzoate, HO* scavenger (HO*: hydroxyl radical). (b) TIRON: 4,5-dihydroxy-1,3-benzene disulfonic acid, $O_2^{\bullet-}$ scavenger ($O_2^{\bullet-}$: superoxide anion). (c) DMTU: N N'-Dimethylthiourea, H_2O_2 scavenger (H_2O_2: hydrogen peroxide). (d) DPI: Diphenyleneiodonium chloride, NADPH oxidase inhibitor (NADPH: nicotinamide adenine dinucleotide phosphate). (e) PCIB: 2-(p-chlorophenoxy)-2-methylpropionic acid, auxin action inhibitor. (f) FLD: Fluridone, ABA biosynthesis inhibitor (ABA: abscisic acid). Chem: a chemical substance added to solution as a scavenger or an inhibitor. Bars indicate the four biological replicates standard error. Different letters above the bars represent significant differences according to Tukey's test (p-value ≤0.05).

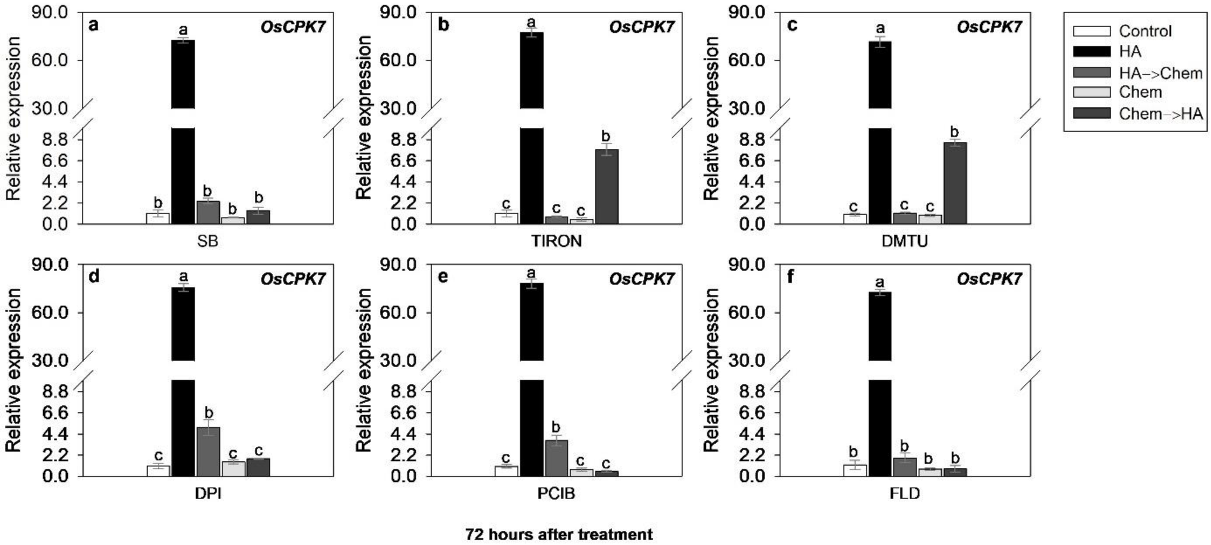

Figure S14. Root development involves an increase in expression level of *OsCPK7* gene, calcium-dependent protein kinase gene family – CDPK, involved in calcium signalling pathways. Rice plants grown for 72 hours in nutrient solution with HA and addition of scavenger or inhibitor (a) SB: sodium benzoate, HO* scavenger (HO*: hydroxyl radical). (b) TIRON: 4,5-dihydroxy-1,3-benzene disulfonic acid, $O_2^{\bullet-}$ scavenger ($O_2^{\bullet-}$: superoxide anion). (c) DMTU: N N'-Dimethylthiourea, H_2O_2 scavenger (H_2O_2: hydrogen peroxide). (d) DPI: Diphenyleneiodonium chloride, NADPH oxidase inhibitor (NADPH: nicotinamide adenine dinucleotide phosphate). (e) PCIB: 2-(p-chlorophenoxy)-2-methylpropionic acid, auxin action inhibitor. (f) FLD: Fluridone, ABA biosynthesis inhibitor (ABA: abscisic acid). Chem: a chemical substance added to solution as an inhibitor or scavenger. Bars indicate the

four biological replicates standard error. Different letters above the bars represent significant differences according to Tukey's test (p-value ≤0.05).

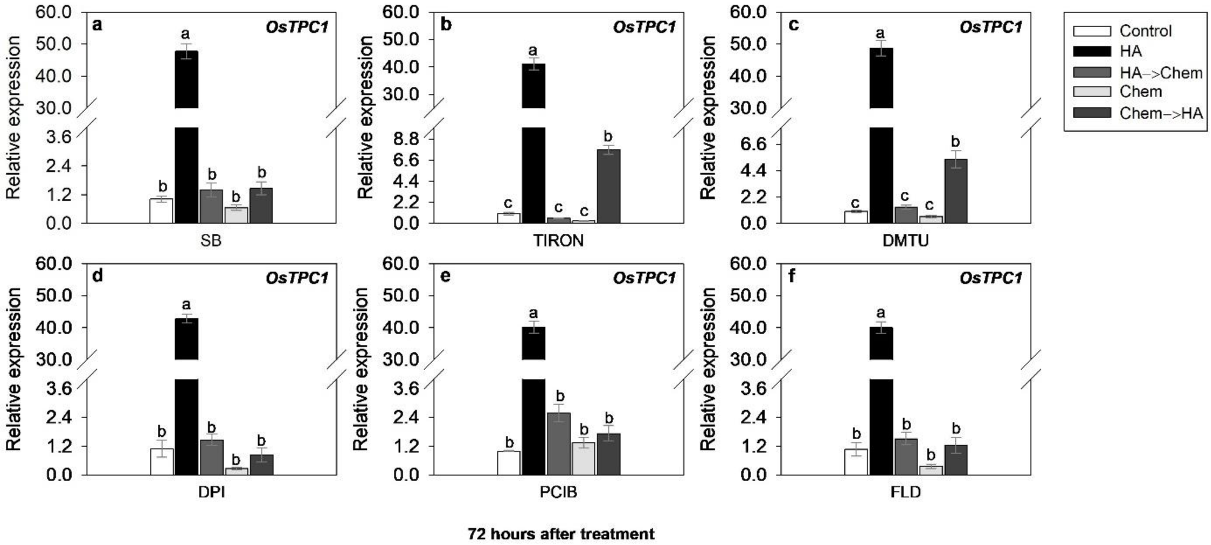

Figure S15. Root development involves an increase in expression level of *OsTPC1* gene, two-pore channel 1, involved in calcium signalling pathways. Rice plants grown for 72 hours in nutrient solution with HA and addition of scavenger or inhibitor (a) SB: sodium benzoate, HO* scavenger (HO*: hydroxyl radical). (b) TIRON: 4,5-dihydroxy-1,3-benzene disulfonic acid, $O_2^{\bullet-}$ scavenger ($O_2^{\bullet-}$: superoxide anion). (c) DMTU: N N'-Dimethylthiourea, H_2O_2 scavenger (H_2O_2: hydrogen peroxide). (d) DPI: Diphenyleneiodonium chloride, NADPH oxidase inhibitor (NADPH: nicotinamide adenine dinucleotide phosphate). (e) PCIB: 2-(p-chlorophenoxy)-2-methylpropionic acid, auxin action inhibitor. (f) FLD: Fluridone, ABA biosynthesis inhibitor (ABA: abscisic acid). Chem: a chemical substance added to solution as an inhibitor or scavenger. Bars indicate the four biological replicates standard error. Different letters above the bars represent significant differences according to Tukey's test (p-value ≤0.05).

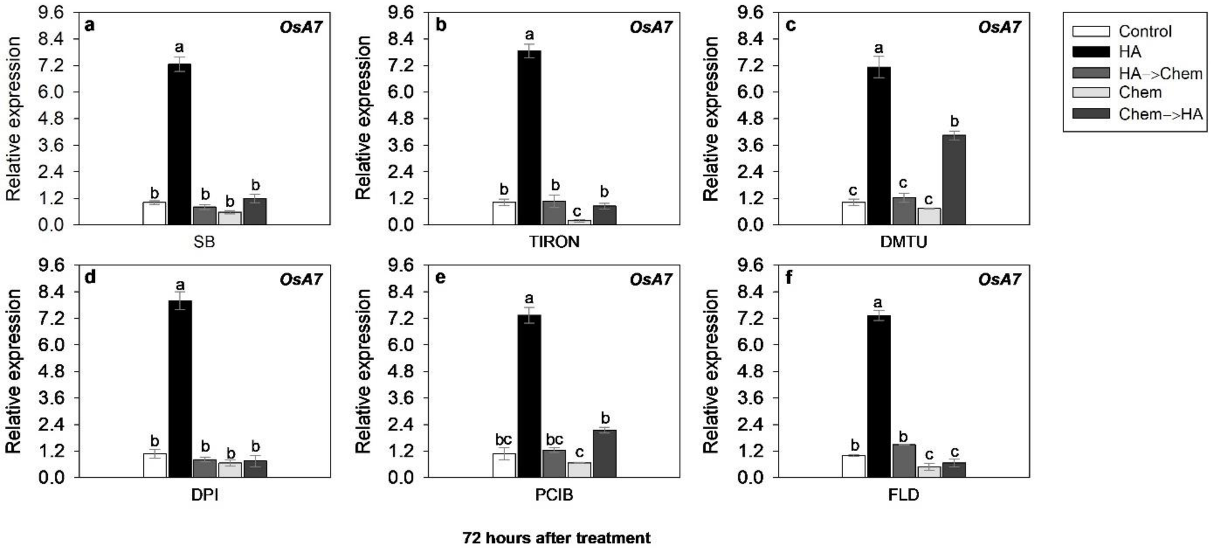

Figure S16. Root development involves an increase in expression level of *OsA7* gene, isoform of plasma membrane H^+-ATPase, *Oryza sativa 7*, involved in PM H^+-ATPase activity and in physiological responses linked to root growth and N uptake. Rice plants grown for 72 hours in nutrient solution with HA and addition of scavenger or inhibitor (a) SB: sodium benzoate, HO* scavenger (HO*: hydroxyl radical). (b) TIRON: 4,5-dihydroxy-1,3-benzene disulfonic acid, $O_2^{\bullet-}$ scavenger ($O_2^{\bullet-}$: superoxide anion). (c) DMTU: N N'-Dimethylthiourea, H_2O_2 scavenger (H_2O_2: hydrogen peroxide). (d) DPI: Diphenyleneiodonium chloride, NADPH oxidase inhibitor (NADPH: nicotinamide adenine dinucleotide phosphate). (e) PCIB: 2-(p-chlorophenoxy)-2-methylpropionic acid, auxin action inhibitor. (f) FLD: Fluridone, ABA biosynthesis inhibitor (ABA: abscisic acid). Chem: a chemical

substance added to solution as an inhibitor or scavenger. Bars indicate the four biological replicates standard error. Different letters above the bars represent significant differences according to Tukey's test (p-value ≤0.05).

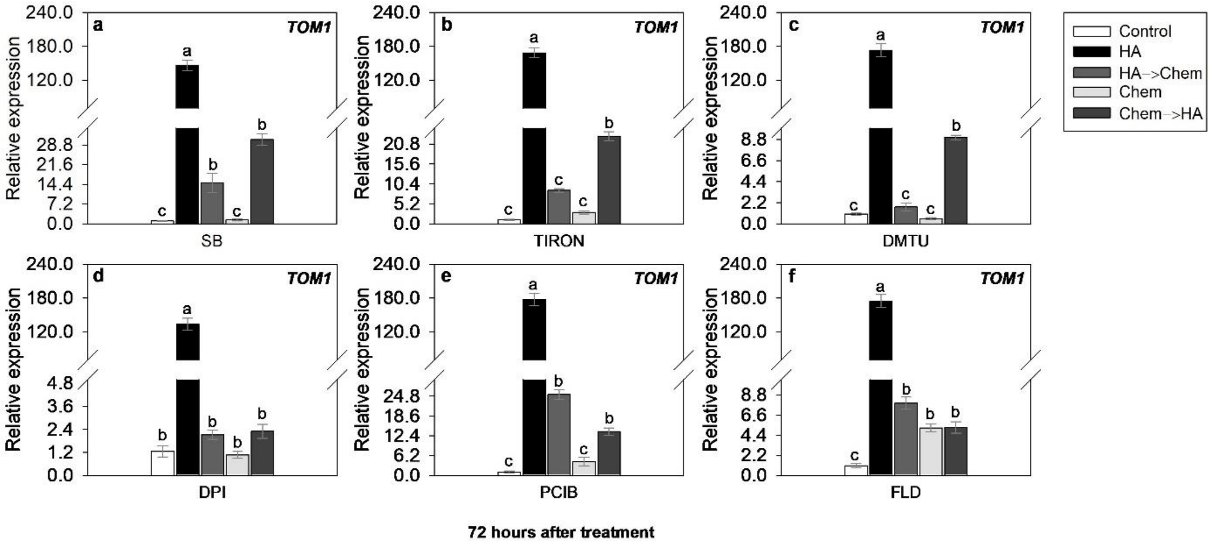

Figure S17. Root development involves an increase in expression level of *TOM1* gene, transporter of mugineic acid 1, involved in regulation of vesicle transport. Rice plants grown for 72 hours in nutrient solution with HA and addition of scavenger or inhibitor (a) SB: sodium benzoate, HO* scavenger (HO*: hydroxyl radical). (b) TIRON: 4,5-dihydroxy-1,3-benzene disulfonic acid, $O_2^{\bullet-}$ scavenger ($O_2^{\bullet-}$: superoxide anion). (c) DMTU: N N'-Dimethylthiourea, H_2O_2 scavenger (H_2O_2: hydrogen peroxide). (d) DPI: Diphenyleneiodonium chloride, NADPH oxidase inhibitor (NADPH: nicotinamide adenine dinucleotide phosphate). (e) PCIB: 2-(p-chlorophenoxy)-2-methylpropionic acid, auxin action inhibitor. (f) FLD: Fluridone, ABA biosynthesis inhibitor (ABA: abscisic acid). Chem: a chemical substance added to solution as an inhibitor or scavenger. Bars indicate the four biological replicates standard error. Different letters above the bars represent significant differences according to Tukey's test (p-value ≤0.05).

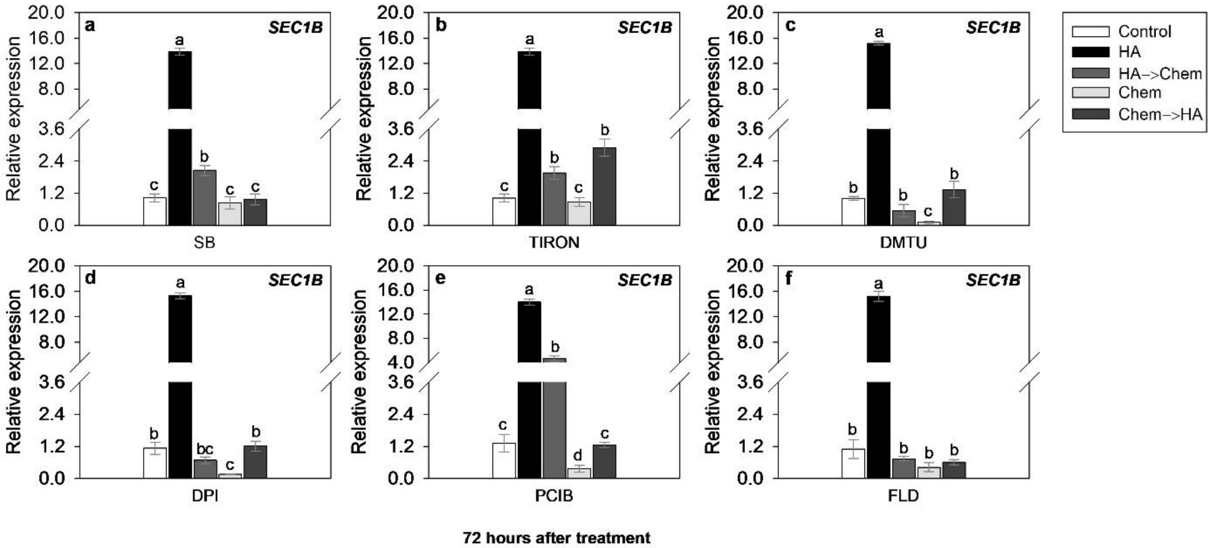

Figure S18. Root development involves an increase in expression level of *SEC1B* gene, secretion 1B – family of protein secretion and transport, involved in regulation of vesicle transport. Rice plants grown for 72 hours in nutrient solution with HA and addition of scavenger or inhibitor (a) SB: sodium benzoate, HO* scavenger (HO*: hydroxyl radical). (b) TIRON: 4,5-dihydroxy-1,3-benzene disulfonic acid, $O_2^{\bullet-}$ scavenger ($O_2^{\bullet-}$: superoxide anion). (c) DMTU: N N'-Dimethylthiourea, H_2O_2 scavenger (H_2O_2: hydrogen peroxide). (d) DPI: Diphenyleneiodonium chloride, NADPH oxidase inhibitor (NADPH: nicotinamide adenine dinucleotide phosphate). (e) PCIB: 2-(p-chlorophenoxy)-2-methylpropionic acid, auxin action inhibitor. (f) FLD: Fluridone, ABA biosynthesis inhibitor (ABA: abscisic acid). Chem: a chemical substance added to solution as an inhibitor or scavenger. Bars indicate the

four biological replicates standard error. Different letters above the bars represent significant differences according to Tukey's test (p-value ≤0.05).

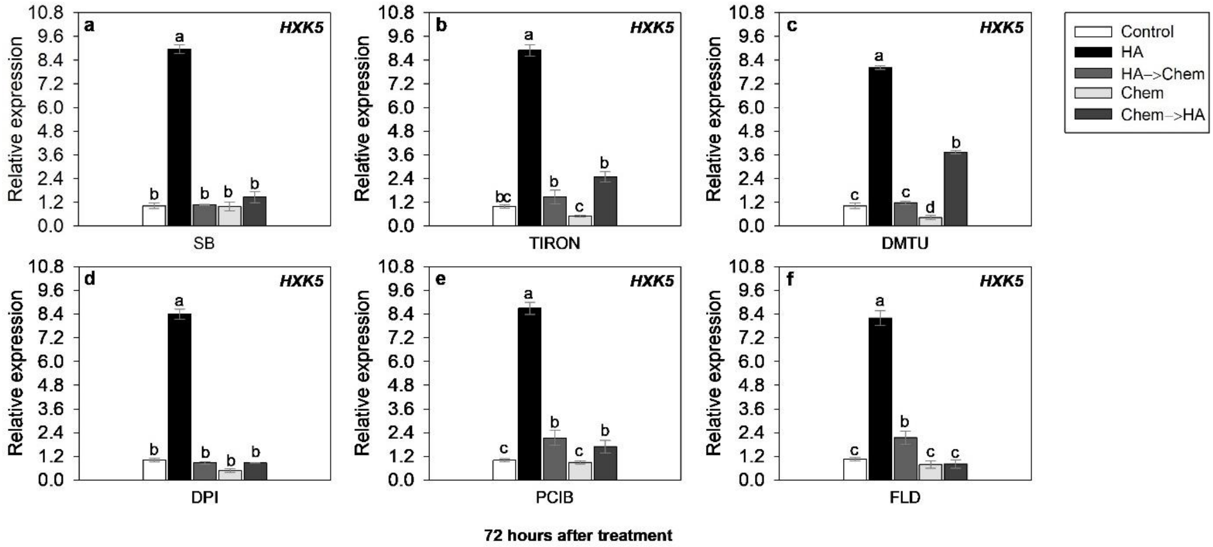

Figure S19. Root development involves an increase in expression level of *HXK5* gene, hexokinase-5, involved in primary metabolism. Rice plants grown for 72 hours in nutrient solution with HA and addition of scavenger or inhibitor (a) SB: sodium benzoate, HO* scavenger (HO*: hydroxyl radical). (b) TIRON: 4,5-dihydroxy-1,3-benzene disulfonic acid, $O_2^{\bullet-}$ scavenger ($O_2^{\bullet-}$: superoxide anion). (c) DMTU: N N'-Dimethylthiourea, H_2O_2 scavenger (H_2O_2: hydrogen peroxide). (d) DPI: Diphenyleneiodonium chloride, NADPH oxidase inhibitor (NADPH: nicotinamide adenine dinucleotide phosphate). (e) PCIB: 2-(p-chlorophenoxy)-2-methylpropionic acid, auxin action inhibitor. (f) FLD: Fluridone, ABA biosynthesis inhibitor (ABA: abscisic acid). Chem: a chemical substance added to solution as an inhibitor or scavenger. Bars indicate the four biological replicates standard error. Different letters above the bars represent significant differences according to Tukey's test (p-value ≤0.05).

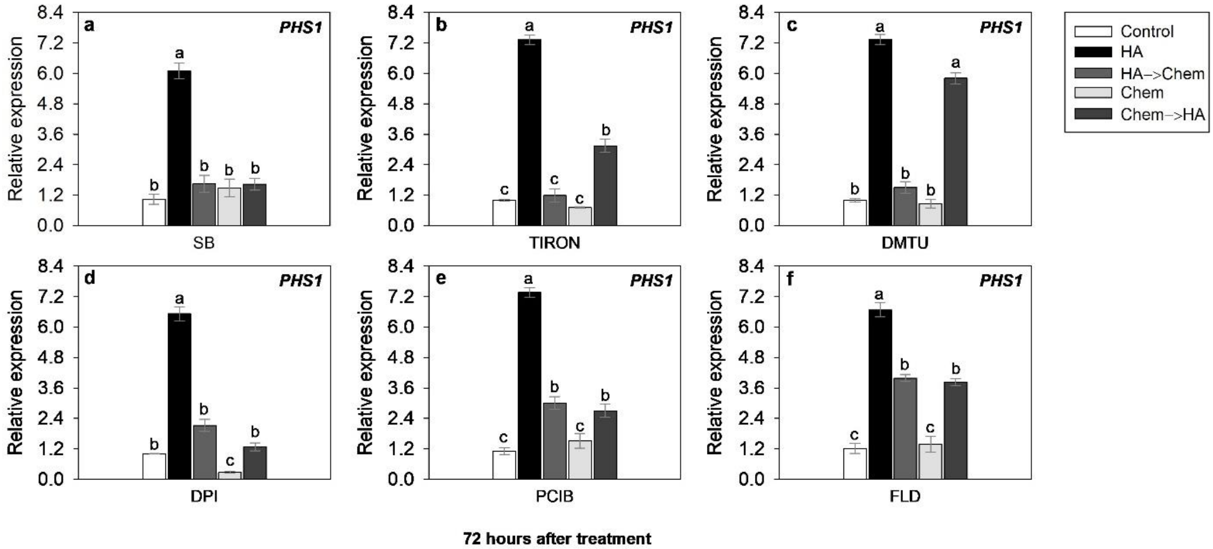

Figure S20. Root development involves an increase in expression level of *PHS1* gene, phosphorylase 1, involved in primary metabolism. Rice plants grown for 72 hours in nutrient solution with HA and addition of scavenger or inhibitor (a) SB: sodium benzoate, HO* scavenger (HO*: hydroxyl radical). (b) TIRON: 4,5-dihydroxy-1,3-benzene disulfonic acid, $O_2^{\bullet-}$ scavenger ($O_2^{\bullet-}$: superoxide anion). (c) DMTU: N N'-Dimethylthiourea, H_2O_2 scavenger (H_2O_2: hydrogen peroxide). (d) DPI: Diphenyleneiodonium chloride, NADPH oxidase inhibitor (NADPH: nicotinamide adenine dinucleotide phosphate). (e) PCIB: 2-(p-chlorophenoxy)-2-methylpropionic acid, auxin action inhibitor. (f) FLD: Fluridone, ABA biosynthesis inhibitor (ABA: abscisic acid). Chem: a chemical substance added to solution as an inhibitor or scavenger. Bars indicate the four biological replicates standard error. Different letters above the bars represent significant differences according to Tukey's test (p-value ≤0.05).

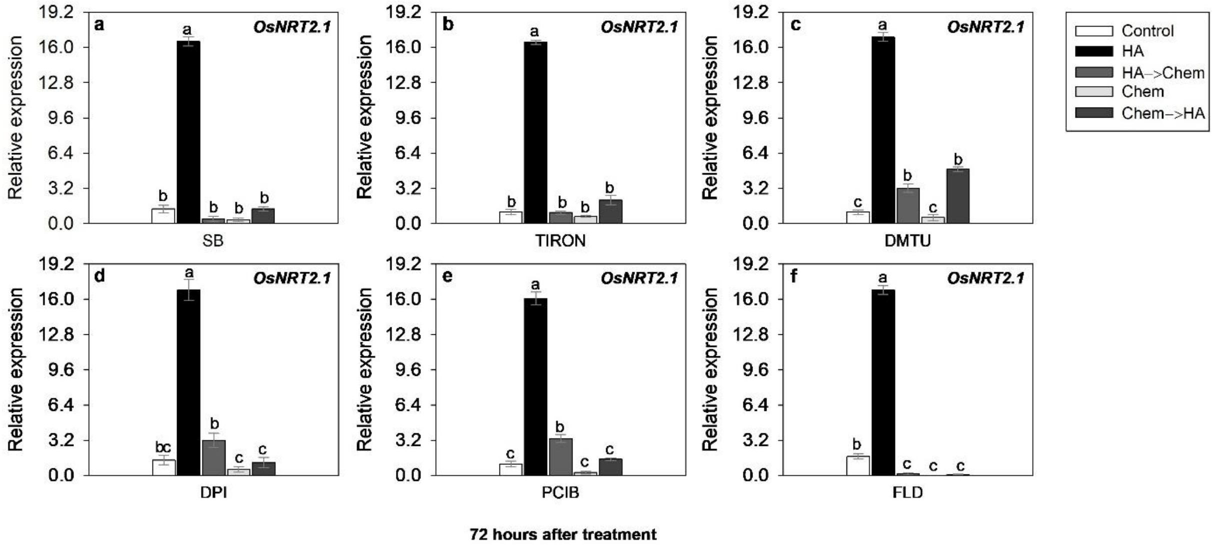

Figure S21. Root development involves an increase in expression level of *OsNRT2.1* gene, nitrate transporter 2.1, involved in physiological responses linked to root growth and N uptake. Rice plants grown for 72 hours in nutrient solution with HA and addition of scavenger or inhibitor (a) SB: sodium benzoate, HO* scavenger (HO*: hydroxyl radical). (b) TIRON: 4,5-dihydroxy-1,3-benzene disulfonic acid, $O_2^{\bullet-}$ scavenger ($O_2^{\bullet-}$: superoxide anion). (c) DMTU: N N'-Dimethylthiourea, H_2O_2 scavenger (H_2O_2: hydrogen peroxide). (d) DPI: Diphenyleneiodonium chloride, NADPH oxidase inhibitor (NADPH: nicotinamide adenine dinucleotide phosphate). (e) PCIB: 2-(p-chlorophenoxy)-2-methylpropionic acid, auxin action inhibitor. (f) FLD: Fluridone, ABA biosynthesis inhibitor (ABA: abscisic acid). Chem: a chemical substance added to solution as an inhibitor or scavenger. Bars indicate the four biological replicates standard error. Different letters above the bars represent significant differences according to Tukey's test (p-value ≤0.05).
